# Effects of a plant cyclotide on conformational dynamics and destabilization of β-amyloid fibrils through molecular dynamics simulations

**DOI:** 10.3389/fmolb.2022.986704

**Published:** 2022-09-30

**Authors:** Neha V. Kalmankar, Bhuvaneshwari Rajendrakumar Gehi, Ramanathan Sowdhamini

**Affiliations:** ^1^ National Centre for Biological Sciences (TIFR), GKVK Campus, Bengaluru, Karnataka, India; ^2^ Molecular Biophysics Unit, Indian Institute of Science, Bengaluru, Karnataka, India; ^3^ Institute of Bioinformatics and Applied Biotechnology, Bengaluru, Karnataka, India

**Keywords:** β-amyloid (Aβ) peptide, disulfide-rich peptides, molecular dynamics (MD) simulation, cyclotides, peptide inhibitors, protein -peptide docking, conformational dynamics, cyclic peptides

## Abstract

Aggregation of β-amyloid (Aβ) peptide is one of the hallmarks of Alzheimer’s disease (AD) which results in chronic and progressive neurodegeneration of the brain. A recent study by our group have shown the ability of cyclic disulfide-rich peptides (“cyclotides”) isolated from a medicinal plant, *Clitoria ternatea*, to inhibit the aggregation of Aβ peptides and reduce oxidative stress caused by reactive oxygen species using *in vivo* models of transgenic *Caenorhabditis elegans*. In the present study, through extensive computational docking and multi-ns molecular dynamics (MD) simulation, we evaluated if cyclotides can stably bind to Aβ molecules and/or destabilize the Aβ fibril by preventing conformational changes from α-helical to β-sheet rich structures. We demonstrate that cyclotides bind effectively and stably to different forms of Aβ structures *via* hydrogen bonding and hydrophobic interactions. One of the conserved hydrophobic interface residues, Tyr10 was mutated to Ala and the impact of this virtual mutation was estimated by additional MD simulations for the wild-type (WT) and mutant protein-peptide complexes. A detailed MD simulation analyses revealed that cyclotides form hydrogen bonds with the toxic amyloid assemblies thereby weakening the inter-strand hydrogen bonds between the Aβ peptide. The φ-ѱ distribution map of residues in the cyclotide binding pocket that ideally adopt β-sheet conformation show deviation towards right-handed ɑ-helical (ɑ_R_) conformation. This effect was similar to that observed for the Tyr10Ala mutant and doubly so, for the cyclotide bound form. It is therefore possible to hypothesise that the opening up of amyloid β-sheet is due to an unfolding process occurring in the Aβ caused by cyclotide binding and inhibition. Our current findings provide novel structural insights on the mode of interaction between cyclotides and Aβ fibrils and describe their anti-amyloid aggregation potential. This sheds light on the future of cyclotide-based drug design against protein aggregation, a hallmark event in many neurodegenerative diseases.

## 1 Introduction

Alzheimer’s disease (AD) is the most common cause of senile dementia and primarily characterized by extracellular plaques of beta-amyloid peptide (Aβ) deposits in the brain (Hardy and Selkoe, 2002). However, the molecular mechanisms for the cause of onset and progression of AD is largely unknown. In all these years, inhibiting Aβ aggregation has become the starting point for therapy against AD as the neurotoxicity is often associated with the pathogenesis of Aβ monomers aggregating into oligomers and fibrils (Jokar et al., 2019). Peptides are a great source for antioxidant properties which can be used therapeutically for the prevention of age-related diseases, specifically as inhibitors of Aβ aggregation in the case of AD (Martorell et al., 2013; Li et al., 2017; Manzanares et al., 2018; Jokar et al., 2020). Additionally, peptides are advantageous over small molecules due to their non-immunogenicity, better bioavailability, low-toxicity and convenient chemical synthesis. In particular, ribosomal synthesized and post-translational modified peptides (RiPP) contain modifications such as cyclization, which improves their ADME properties (absorption, distribution, metabolism, and excretion). Such modifications can improve the oral bioavailability and ability to pass the blood-brain-barrier, which still remains as a major challenge of peptide-based drug development.

Cyclotides are disulfide-rich mini-proteins found in plants with a unique head-to-tail cyclized backbone and three disulfide bonds forming a cyclic cystine-knot motif. This circularised knotted arrangement makes cyclotides remarkably stable against enzymatic, chemical and thermal degradation (Daly et al., 2009). Few years ago, synthetically created cyclotides, analogous to the *Oldenlandia affinis* plant-derived peptide, could be orally administered in animal models to suppress multiple sclerosis (MS), an autoimmune disease affecting the central nervous system (Thell et al., 2016). More recently, the anti-neurodegenerative properties of cyclotides were studied in *Psychotria solitudinum*, where they acted as inhibitors of the human prolyl oligopeptidase, a promising target for the treatment of cognitive deficits in several psychiatric and neurodegenerative diseases (Hellinger et al., 2015). Similarly, Wang and co-workers demonstrated that naturally occurring cyclic peptides, sunflower trypsin inhibitor 1 (SFTI-1) and the cyclotide kB1, have an inherent ability to inhibit tau fibril growth and aggregation, specifically the AcPHF6—a tau-derived peptide (Wang et al., 2016). Our group recently demonstrated the *in vivo* anti-Aβ effects of cyclotides, purified from a Indian medicinal plant *Clitoria ternatea,* using three transgenic *Caenorhabditis elegans* models that exhibits pathological behaviours associated with Aβ (Kalmankar et al., 2021). We described a novel neuroprotective activity of cyclotides against Aβ-induced toxicity indicating that these could be potential therapeutic leads for blocking the progression of AD. The current study describes the usage of molecular docking analyses and molecular dynamics (MD) simulations to establish the molecular and atomic level interactions between Aβ protofibril structures and cyclotide.

We evaluated if cyclotides can stably bind to Aβ and/or destabilize both the α-helical and β-sheet rich structures of Aβ fibrils by causing conformational changes using molecular dynamics (MD) simulation. We utilized four different polymorphs of, such as the Aβ_1-42_ monomer (PDB ID: 1IYT) (Crescenzi et al., 2002), U-shaped pentamer Aβ_17-42_ (PDB ID: 2BEG) (Lührs et al., 2005), S-shaped model Aβ_11-42_ (PDB ID: 2MXU) (Xiao et al., 2015) and a disease relevant Aβ_1-42_ fibrils (PDB ID: 2NAO) (Wälti et al., 2016) to capture the potential of cyclotides to bind to Aβ molecules at different stages of their growth. Apart from carrying out MD simulations for the wild-type (WT) Aβ fibril, we also performed 300 ns MD simulations on a Y10A mutant conformation of the disease relevant form of Aβ_1-42_ (PDB ID: 2NAO), to investigate their structural and dynamical properties upon cyclotide binding. The single tyrosine residue at position 10 of the Aβ is a typical aromatic amino acid in the hydrophilic segment of Aβ and implicated in stabilization of Aβ aggregation (Barnham et al., 2004; Dai et al., 2012). Tyr10 is also reported to be active towards copper and heme-binding, thereby being an important site for post-translational and chemical modifications such as phosphorylation and nitration of Aβ (Atwood et al., 2004; Lu et al., 2015). Our results show that cyclotides have a potent ability to bind and weaken Aβ fibril aggregation and combined with the *in silico* single point-mutation, they tend to have a more profound destabilizing effect. We analysed the φ-ѱ distribution on the Ramachandran map for residues adopting parallel β-sheet conformation of the cyclotide binding pocket and the results clearly show that significant deviation occurs from ideal dihedral angles of β-sheet conformation to right-handed ɑ-helical (ɑ_R_) region. Therefore, it is highly probable that this β-sheet to ɑ_R_ transition is due to an unfolding process occurring in the Aβ molecules triggered by cyclotide binding.

Overall, our findings from the current study provides novel insights on the potential of cyclotides as Aβ inhibitors, specifically elucidating the molecular mechanisms involved in destabilization of Aβ protofibrils. Together with our *in vivo* data involving transgenic *Caenorhabditis elegans* model of Alzheimer’s disease, the computational results presented in this study provide compelling evidence that cyclotides have anti-amyloid aggregation properties and can act as an important source of drug leads for inhibiting fibril formation.

## 2 Materials and methods

### 2.1 Protein—peptide docking

In order to better understand and visualize the mode of interactions between different forms of Aβ fibrillar species and cyclotide, we performed molecular docking experiments using FRODOCK2.0 (Ramírez-Aportela et al., 2016). The four different three-dimensional (3D) NMR solution structures of Aβ used in this study are: 1) PDB ID: 1IYT, Aβ_1-42_ monomer (Crescenzi et al., 2002), 2) PDB ID: 2BEG, U-shaped pentamer Aβ_17-42_ (Lührs et al., 2005), 3) PDB ID: 2MXU, S-shaped model Aβ_11-42_ (Xiao et al., 2015) and 4) PDB ID: 2NAO, disease relevant Aβ_1-42_ fibrils (Wälti et al., 2016). We used the three-dimensional NMR solution structure of Cter-M cyclotide from *C. ternatea* (PDB ID: 2LAM) (Poth et al., 2011) to dock against each of the 4 Aβ fibrillar species. For all the generated docked poses for each of the cyclotide-Aβ complexes (4 complexes), PPCheck was used to calculate binding energies and to predict best native-like docking pose (Sukhwal and Sowdhamini, 2015).

### 2.2 Molecular dynamics simulation of WT and mutant structures

#### 2.2.1 Protein preparation

The mutant structure (Y10A of chain C) of the Aβ was generated from the original PDB structure (PDB ID: 2NAO) in the Maestro package (Schrödinger Release 2020: Maestro, Schrödinger, LLC, New York, NY, 2020). The four wild-type Aβ-cyclotide complexes (1IYT-2LAM, 2BEG-2LAM, 2MXU-2LAM and 2NAO-2LAM) and the Y10A mutant Aβ-cyclotide complex (2NAO-2LAM) was minimized at pH 6.8 using PROPKA from Protein Preparation Wizard (Sastry et al., 2013). Each structure was restrain minimized using the OPLS3e force field (Harder et al., 2015).

#### 2.2.2 Protein solvation

Each complex was solvated with TIP4P solvent model with an orthorhombic box for boundary conditions and buffer distance of 10 Å using System Builder option of the Desmond module of Schrodinger (Bowers et al., 2006). The system was minimized using steepest descent for 2000 steps until a gradient threshold of 50 kcal mol^−1^ Å^−1^ was reached. The system was neutralised with either Na^+^ or Cl^−^ ions and additional 150 mM NaCl salt was added. The output from the system builder was used for MD simulations.

#### 2.2.3 MD simulations

To confirm stable binding of cyclotide to each wild-type Aβ and mutant (Y10A) Aβ conformation, triplicate runs of MD simulations without constraints was performed using the Desmond package of Maestro Schrodinger (Bowers et al., 2006). The output from System Builder option was the input for MD simulations and three independent simulation runs with different random initial velocities, each of them 300 ns long, was performed. Triplicate set of control MD runs were also performed on each of the unbound WT and mutant Aβ structure. The simulation time was set at 300 ns in the NPT ensemble class for each simulation. As commonly observed in literature, MD simulations are performed for 100–300 ns when dealing with small proteins and 1 µs simulations are usually performed for large proteins or receptors. As the molecules of interest in the present study are both peptides, we have used 300 ns timeframe for the simulations. The RESPA integrator was used with a time step of 2.0 fs (Humphreys et al., 1994). The temperature and pressure were set at 300 K and 1 bar, using the Nose-Hoover chain (Martyna et al., 1992) and the Martyna-Tobias-Klein method (Martyna et al., 1994) respectively.

### 2.3 Conformational analysis of protein—peptide complex

For stability and conformational analyses, the entire range of simulation time of each MD trajectory was considered. The root mean square deviation (RMSD), root mean square fluctuation (RMSF), radius of gyration (Rg) and number of hydrogen bonds was calculated for each trajectory of all the four WT Aβ (protein)—cyclotide (peptide) complexes and mutant Aβ-cyclotide complex using the Simulation Event Analysis (SEA) module implemented in the Desmond package. Additionally, protein-peptide interactions were also monitored throughout the simulation time using the Maestro package. For computing the energy terms, following a similar methodology as Roy et al. (Roy et al., 2022), the total energy values (kcal/mol) were collected at 10 ps intervals across the 300 ns MD simulation trajectory (a total of 30,000 timestamps) for WT Aβ_1-42_—cyclotide complex and its control trajectory (WT Aβ_1-42_ unbound), and mutant Aβ_1-42_ (Y10A)—cyclotide complex and its control trajectory (Aβ_1-42_ Y10A unbound) using the Simulation Quality Analysis (SQA) module in the Desmond package. The difference in total energy (ΔE) between the cyclotide bound and unbound forms along the time series (i; 10 ps intervals) was calculated using the formulae:

$$\Delta E_{WT\left(i\right)}=E_{WT-bound\left(i\right)}-E_{WT-unbound\left(i\right)}$$

$$\Delta E_{Y10A\left(i\right)}=E_{Y10A-bound\left(i\right)}-E_{Y10A-unbound\left(i\right)}$$

## 3 Results

### 3.1 Cyclotide binding to different polymorphs of Aβ

**FIGURE 1:**
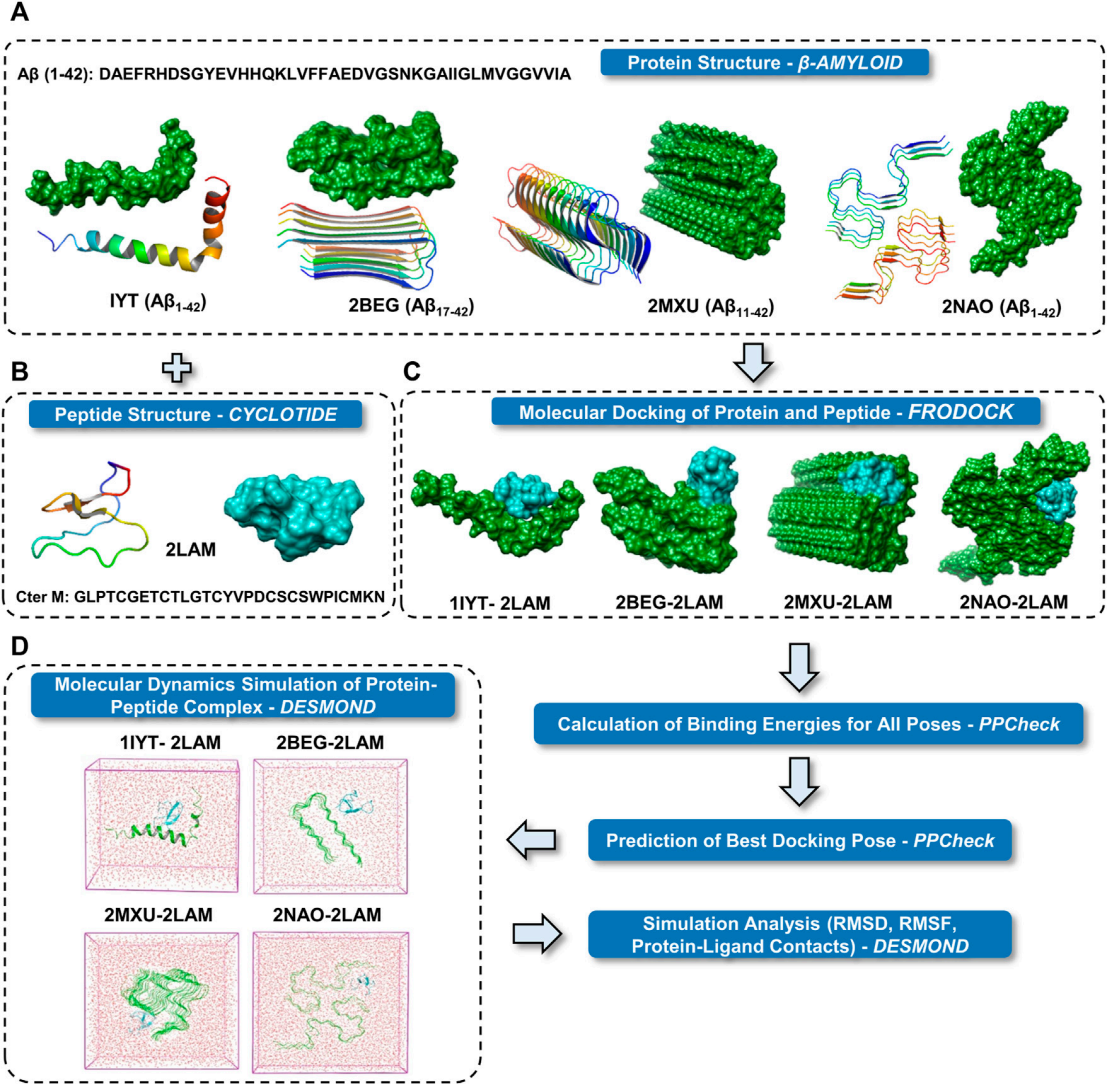
Schematic representation of the research protocol used in the present study. **(A)** Cartoon and surface representations of the four different Aβ structures. **(B)** Cartoon and surface representation of cyclotide Cter M structure. **(C)** Surface representations of the best binding mode of the four docked complexes of Aβ and cyclotides. Color scheme used for surface representation: green—Aβ and cyan—cyclotide. **(D)** The protein complexes are minimized and placed in a box of water. The systems were then set up for MD simulation.

A general schema for docking and MD simulation of the multi-protein complexes employed in the present study is described in Figure 1. Two most common isoforms of Aβ are Aβ_1-40_ and Aβ_1-42_ depending on the position of cleavage on the amyloid precursor protein (APP). Several polymorphs of amyloid structures exist and plenty of high-resolution structures are available for Aβ_1-40_ fragments. However, high resolution three-dimensional structures of the disease relevant form of Aβ are relatively few. We have included the NMR derived hexameric S-shaped structure of full length Aβ_1-42_ (PDB ID: 2NAO) as a representative amyloid structure to understand in detail the possible interaction of Aβ fibril and cyclotide. Nevertheless, there is high complexity and diversity in the amyloid folds and in order to span across the ability of cyclotide to bind to different polymorphs, we also included three other Aβ quaternary structures for molecular docking and subsequent MD simulations (the results for which are in Supplementary Material). These include: PDB ID 1IYT (Aβ_1-42_ monomer), PDB ID 2BEG, (U-shaped pentamer Aβ_17-42_) and PDB ID 2MXU (S-shaped model Aβ_11-42_) (Figure 1A). Of these three NMR models, one is a monomeric form of Aβ_1-42_ (PDB ID: 1IYT) and the other two are fragments of the fibrillary form (PDB IDs: 2BEG and 2MXU). Only one NMR structure of chemically synthesized Cter-M cyclotide from *C. ternatea* (PDB ID 2LAM) is known till date and we have used it as a representative cyclotide structure for all the docking and simulation studies (Figure 1B) (Poth et al., 2011). To evaluate the molecular interactions and binding affinities, we carried out rigid-body docking studies using FRODOCK2.0 between cyclotide and different forms of Aβ peptide (Figure 1C) (Ramírez-Aportela et al., 2016). FRODOCK output comprising of top 10 poses for each of protein-peptide complex were evaluated using our in-house program PPCheck to predicting the best docking pose based on the total stabilizing energy and normalized energy per residue (Sukhwal and Sowdhamini, 2015). PPCheck quantifies the strength of protein-protein interaction using pseudo-energies as van der Waals, electrostatic and hydrogen bonds. For 1IYT-2LAM, 2BEG-2LAM, 2MXU-2LAM and 2NAO-2LAM complexes, pose-2, 3, 7 and 10 were the best-docked poses, respectively (Supplementary Table S1). From the PPCheck analyses, the pose with lowest normalized energy per residue were selected to perform MD simulations (Figure 1D).

### 3.2 Molecular dynamics simulation analyses of wild-type Aβ-cyclotide complex

**FIGURE 2:**
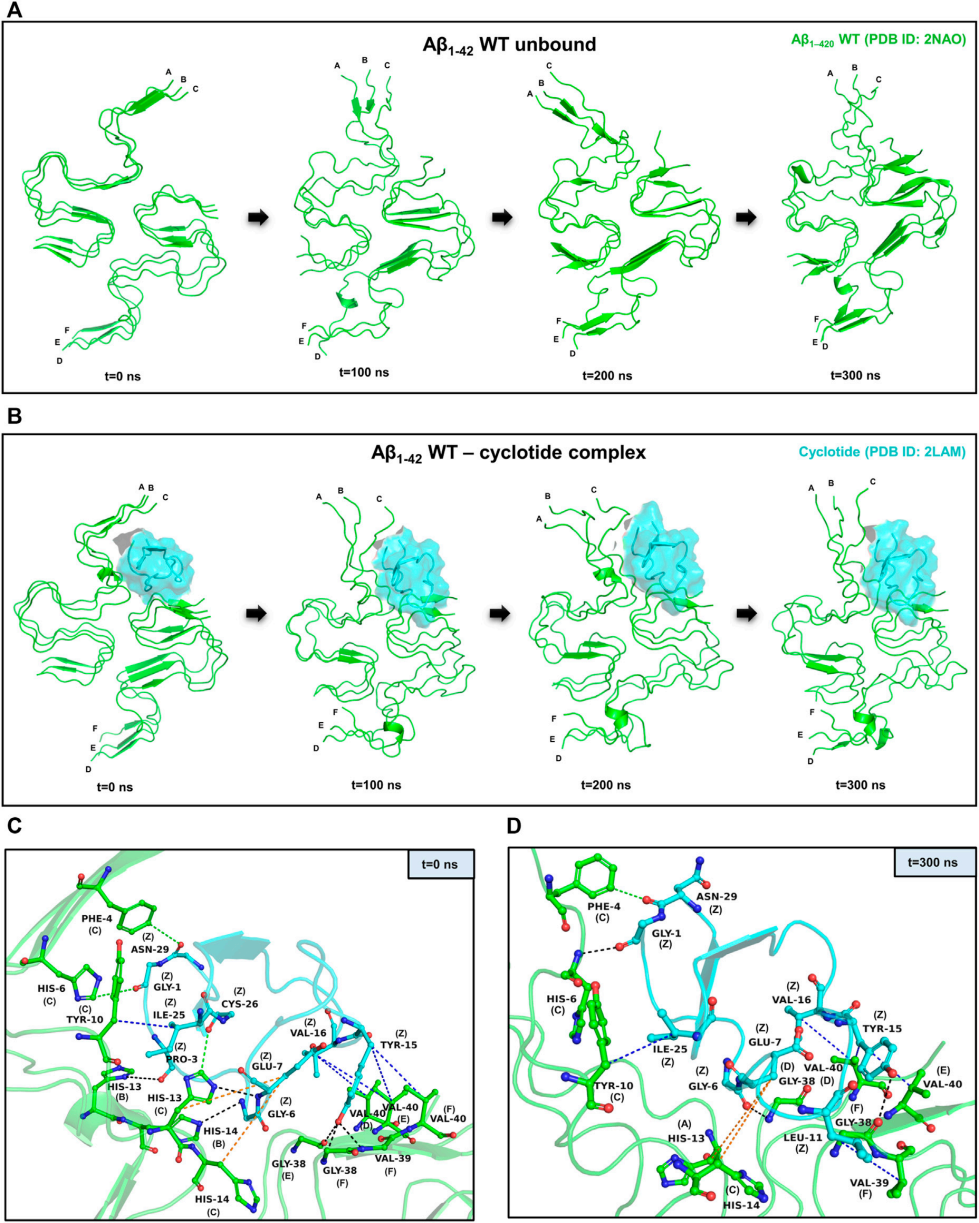
Representative example of MD simulations between the wild-type Aβ_1-42_ fibril and cyclotide Cter-M. **(A)** Snapshots of MD simulation of unbound Aβ_1-42_ fibril (PDB ID: 2NAO; green coloured cartoon representation) at different time points along the simulation period. **(B)** Snapshots of MD simulation of WT Aβ_1-42_ fibril (PDB ID: 2NAO; green coloured cartoon representation) and Cter-M (PDB ID: 2LAM; cyan surface representation) complex at different time points along the simulation period. Capital letters, which denote the polypeptide chains of 2NAO, are placed at the 1st residue of each chain. **(C,D)** Molecular interactions between the WT Aβ_1-42_ fibril (PDB ID: 2NAO; chains A-F; green cartoon representation) and cyclotide Cter-M (PDB ID: 2LAM; chain Z; cyan cartoon representation) at the beginning (0^th^ ns snapshot) and end (300^th^ ns snapshot) of the simulation period, respectively. Colour scheme for interactions used: classical hydrogen bonds in black, aromatic hydrogen bonds in green, hydrophobic interactions in blue and electrostatic interactions in orange.

To assess the stability of the docked poses for each protein-peptide complex, molecular dynamics (MD) simulations were carried out using the Desmond package of Maestro Schrodinger (Bowers et al., 2006). The Aβ_1-42_ fragment is the dominant Aβ species compared to other polymorphs in the amyloid plaques of AD and hence, we used the hexameric structure (PDB ID: 2NAO) of Aβ_1-42_ fibril as a representative for detailed analysis of structural and dynamical changes that occur upon cyclotide binding to Aβ. We performed three independent 300 ns MD simulations for cyclotide (PDB ID: 2LAM) in complex with the wild-type Aβ_1-42_ fibril (PDB ID: 2NAO). The averaged backbone root mean square deviations (RMSD), root mean square fluctuation (RMSF) and radius of gyration (Rg) of the Aβ_1-42_ with and without cyclotides were calculated as a function of time. Between the bound and unbound forms of Aβ, there were greater fluctuations seen in the cyclotide-bound Aβ structure compared to unbound Aβ. The stability of the cyclotide Cter-M interaction with the disease relevant Aβ_1-42_ fibril is highlighted in Figure 2. It is evident that upon binding to Cter-M, Aβ_1-42_ fibril undergoes large conformational changes and the secondary structures, especially extended β-sheet conformations, begin to disrupt soon after 50 ns. This is in contrast to the unbound Aβ_1-42_ fibril, without the cyclotide molecules, wherein extended β-sheet conformations are relatively more stable throughout the simulation trajectory (Figures 2A,B). If we observe the changes in secondary structures (Figures 2, 4), greater deviations in extended β-sheet configuration was observed in cyclotide-bound Aβ_1-42_ complex than unbound forms. These deviations were not just detected at the cyclotide binding regions but also at other β-sheet regions in the fibril, which are known to be the molecular determinants of fibrillar assembly. We believe the reason for these secondary structural changes upon cyclotide binding, is due to the sequence, size and highly constrained scaffold (three disulfide bonds forming a knotted fold) of the cyclotide. In a study by Wang et al., it was observed that kalata B1, a cyclotide from *Oldenlandia affinis*, showed stronger inhibition to the AcPHF6 fibril formation than SFTI-1, another naturally occurring, disulfide-rich, cyclic peptide, suggesting that the sequence and structure of kB1 makes it better at disrupting the fibrils (Wang et al., 2016). Additionally, unlike the S-shaped Aβ_1-42_ fibril, the cyclotides’ secondary structure remains stable throughout the simulations. The stability of cyclotides is crucial to their natural activity, and this stability inherently comes from the disulfide knot. The timeline analysis of the secondary structural variations during the 300 ns MD simulations shows that extended β-sheet conformations (in yellow) were stable throughout the trajectory in the unbound Aβ_1-42_ fibril (Supplementary Figure S1A). However, in the cyclotide-Aβ_1-42_ bound trajectory, the binding pocket region undergoes significant structural changes soon after 50 ns and loses the extended β-sheet conformations, especially around residues 2–7 (chains A-C) and 38–41 (chains D-F) of Aβ_1-42_ structure (Supplementary Figure S1B). The backbone RMSD, RMSF, radius of gyration and hydrogen bond parameters in each of the triplicate MD trajectories show that the complexes stabilize soon after 50–60 ns and remain stable throughout the simulation time (Supplementary Figure S2).

PPCheck and Maestro package was utilized for identifying all the possible protein-protein interactions including classical hydrogen bonds, aromatic hydrogen bonds, electrostatic and hydrophobic interactions, salt bridges and π-π interactions. Figures 2C,D summarizes the molecular interactions between the Aβ_1-42_ fibril and Cter-M at the beginning (0^th^ ns snapshot) and end (300^th^ ns snapshot) of the simulation period, respectively. At t = 0 ns, the Aβ_1-42_ fibril and Cter-M complex is mainly stabilized by six hydrogen bonds, three aromatic hydrogen bonds, five hydrophobic interactions and two electrostatic interactions (Figure 2C; Supplementary Table S2). The interaction interface involves polar and aromatic residues of Aβ i.e., His13, His14 from chain B, Phe4, His6, Tyr10, His13 and His14 from chain C and aliphatic residues such as Val40 from chain D, Gly38 and Val40 from chain E, and Gly38, Val39 and Val40 from chain F. The residues of Cter-M involved in the interaction are Gly1, Pro3, Gly6, Glu7, Tyr15, Val16, Ile25, Cys26, and Asn29. At t = 300 ns, the Aβ_1-42_ fibril and Cter-M complex is mainly stabilized by three hydrogen bonds, one aromatic hydrogen bonds, four hydrophobic interactions and two electrostatic interactions (Figure 2D; Supplementary Table S2). The interaction interface at the end of 300 ns simulations involves similar residues of Aβ as mentioned earlier i.e., His13 from chain A, Phe4, His6, Tyr10, His14 from chain C, and aliphatic residue such as Gly38 from chain D and F, Val39 from chain F and Val40 from chain D and E. Cter-M residues stabilizing the Aβ_1-42_ are also comparable comprising of Gly1, Gly6, Glu7, Leu11, Tyr15, Val16, Ile25 and Asn29. The total stabilizing energetics at t = 0 ns and 300 ns frames for the cyclotide—Aβ_1-42_ fibril complex is detailed in Table 1. The intermolecular hydrogen bonds were also monitored throughout the simulation period as these are relative measures of binding affinity. An average number of 3-4 hydrogen bonds are present in the cyclotide—Aβ_1-42_ fibril complex (Supplementary Figure S2).

**TABLE 1:** The total stabilizing energy at t = 0 ns and t = 300 ns for the disease relevant wild-type Aβ_1-42_ fibril (PDB ID: 2NAO) interacting with cyclotide Cter-M (PDB ID: 2LAM).

Total stabilizing energy	0^th^ ns frame	300^th^ ns frame
Hydrogen Bond Energy (kJ/mol)	−20.12	−25.54
Electrostatic Energy (kJ/mol)	−4.65	−2.59
Van der Waals Energy (kJ/mol)	−208.13	−184.12
Total Stabilizing Energy (kJ/mol)	−232.91	−212.25
Number of interface residues	75	75
Normalized Energy per residue (kJ/mol)	−3.11	−2.83
No. of Hydrophobic Interactions	5	5
No. of van der Waals Pairs	7,768	7,687
No. of Salt Bridges	0	0
No. of Potential Favourable Electrostatic Interactions	2	2

Cyclotide has the potential to stably bind and disrupt the ordered structures of several forms of amyloid fibrils and the interactions remain stable throughout (Supplementary Figures S3, S4). As evident from the plot of RMSD variation, all systems experienced some degree of fluctuations at first, but gradually tended to converge after 60 ns implying that the simulations reached equilibrium. The intermolecular hydrogen bonds in the remaining three protein-peptide complexes were also monitored throughout the simulation period. The average number of hydrogen bonds between complexes 1IYT-2LAM, 2BEG-2LAM and 2MXU-2LAM were 3, 1 and 3, respectively (Supplementary Figure S4). The total stabilizing energy at t = 0 ns and t = 300 ns frames for the three cyclotide—Aβ complexes is detailed in Supplementary Table S3. Molecular interactions between the three other Aβ polymorphs and cyclotides are illustrated in Supplementary Figure S5 and Supplementary Tables S4–6.

### 3.3 Molecular dynamics simulation analyses of Y10A mutant Aβ-cyclotide complex

In order to analyse the structural consequences of cyclotide binding upon mutation in the Aβ, an *in silico* single point-mutation was created at position 10 in chain C of the Aβ_1-42_ fibril (PDB ID: 2NAO) structure, which originally contains a tyrosine residue at this position. We selected this S-shaped Aβ_1-42_ fibrillar structure with a dimer base (PDB ID: 2NAO), deduced from solid-state NMR experiments and electron microscopy mass-per-length measurements, as a representative model for *in silico* mutation, as it is believed to represent the *in vivo* form of the mature Aβ fibrils, i.e., disease-relevant polymorph consisting of mainly extended configurations of β-sheets and β-turns (Wälti et al., 2016; Wang et al., 2018). Also, among the four selected Aβ structures in the present study, only 2NAO contains the full sequence of Aβ. The tyrosine at position 10 was mutated to alanine (Y10A) and the constructed structure was subjected to 300 ns of MD simulations. Our reason to choose this site for *in silico* mutagenesis was based on the fact that Tyr10 has an important role in stabilization of Aβ aggregation. Moreover, it is present as part of the hydrophobic core and yet within interacting distance with the cyclotide. Many reports also suggest that a mutation at Tyr10 position can prevent Aβ aggregation and fibril growth, thereby highlighting it as a key site for drug targeting (Barnham et al., 2004; Dai et al., 2012; Lu et al., 2015; Jiang et al., 2019).

**FIGURE 3:**
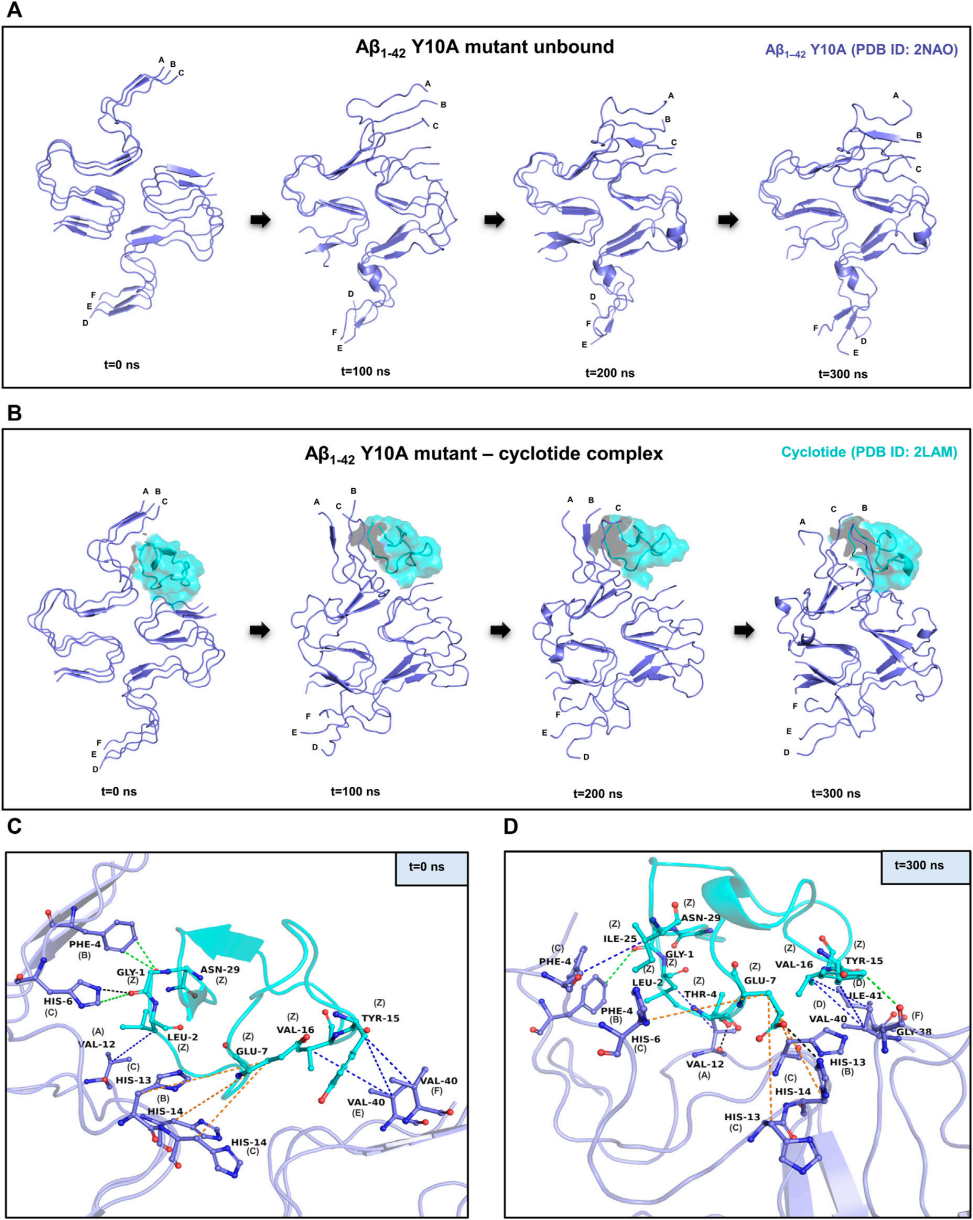
MD simulations between the Y10A mutant conformation of Aβ_1-42_ and cyclotide Cter-M. **(A)** Snapshots of MD simulation of unbound Y10A mutant form of Aβ_1-42_ (PDB ID: 2NAO; green coloured cartoon representation) at different time points along the simulation period. **(B)** Snapshots of MD simulation of Y10A mutant form of Aβ_1-42_ (PDB ID: 2NAO; green coloured cartoon representation) and Cter-M (PDB ID: 2LAM; cyan surface representation) complex at different time points along the simulation period. Capital letters, which denote the polypeptide chains of 2NAO, are placed at the 1^st^ residue of each chain. **(C,D)** Molecular interactions between the Y10A mutant Aβ_1-42_ fibril (PDB ID: 2NAO; chains A-F; green cartoon representation) and cyclotide Cter-M (PDB ID: 2LAM; chain Z; cyan cartoon representation) at the beginning (0^th^ ns snapshot) and end (300^th^ ns snapshot) of the simulation period, respectively. Colour scheme for interactions used: classical hydrogen bonds in black, aromatic hydrogen bonds in green, hydrophobic interactions in blue and electrostatic interactions in orange.

Similar to the wild-type, it is evident in the Y10A mutant Aβ_1-42_—cyclotide complex, that the Aβ fibril undergoes significant conformational changes upon binding to Cter-M. The secondary structures formed by mainly extended parallel β-sheets begin to disrupt soon after 50 ns. However, unlike the wild-type Aβ_1-42_, the unbound form of Aβ_1-42_ Y10A mutant seems to undergo structural changes throughout the simulation trajectory (Figures 3A,B). This is not surprising, as it is known that Tyr10 is an important site for the maintenance of Aβ aggregation and a mutation at this position causes instability in the well-ordered structure of Aβ molecules. Supplementary Figure S6A highlights the secondary structural variations in Y10A mutant Aβ_1-42_ unbound and cyclotide bound forms, during the simulation period. It is evident that in the unbound form of Aβ_1-42_ Y10A mutant, extended β-sheet conformations (in yellow) are disrupted throughout the trajectory, highlighting the importance of Tyr10 for Aβ structural integrity. However, in the Y10A mutant Aβ_1-42_—cyclotide complex (Supplementary Figure S6B), the destabilization caused by both the Y10A mutation and cyclotide binding is significantly drastic, as the secondary structures of the binding pocket region (residues 2-7 of chains A-C and residues 38–41 of chains D-F) undergo deviations by losing the extended β-sheet conformations throughout the simulation trajectory. The stability of the structural complexes was also assessed by plotting the backbone RMSD, RMSF and radius of gyration, and the plots show that the complexes remain stable throughout the simulation time and an average number of 3-4 hydrogen bonds are present in the cyclotide—Aβ_1-42_ (Y10A mutant) fibril complex (Supplementary Figure S2). The stability of the structural complexes was assessed by plotting the backbone RMSD, RMSF and radius of gyration. The plots show that the complexes remain stable throughout the simulation time and an average number of 3-4 hydrogen bonds are present in the cyclotide—Aβ_1-42_ (Y10A mutant) fibril complex (Supplementary Figure S2).

Figures 3C,D summarizes the molecular interactions between the Y10A mutant form of Aβ_1-42_ and Cter-M at the beginning (0^th^ ns snapshot) and end (300th ns snapshot) of the simulation period, respectively. It is evident that the strength of interaction at the interface of the mutant Aβ and cyclotide seems to have reduced significantly as compared to the wild-type. Only a few residues are observed to involved in contacts between the Y10A mutant Aβ_1-42_ structure and the cyclotide (Figures 2C,D, 3C,D; Supplementary Tables S2, S7). At t = 0 ns, the Y10A mutant Aβ_1-42_ and Cter-M complex is mainly stabilized by one hydrogen bond, three aromatic hydrogen bonds, four hydrophobic interactions and three electrostatic interactions (Figure 3C; Supplementary Table S7). The interaction interface is similar to the WT Aβ-cyclotide complex involving polar and aromatic residues of Aβ i.e., Phe4 and His14 from chain B, His6, His13 and His14 from chain C, and aliphatic residues such as Val12 from chain A, and Val40 from chain E and chain **(F)**. The residues of Cter-M involved in the interaction are Gly1, Leu2, Glu7, Tyr15, Val16 and Asn29. At t = 300 ns, the Aβ_1-42_ fibril and Cter-M complex is mainly stabilized by two hydrogen bonds, two aromatic hydrogen bonds, five hydrophobic interactions and four electrostatic interactions (Figure 3D; Supplementary Table S7). The interaction interface at the end of 300 ns simulations involves similar network of residues as mentioned earlier, i.e., Val12 from chain A, Phe4 and His13 from chain B, Phe4, His6, His13, His14 from chain C, and aliphatic residue such as Gly38 from chain F, Val40 and Ile41 from chain **(D)**. Cter-M residues stabilizing the Aβ_1-42_ are also comparable comprising of Gly1, Leu2, Thr4, Glu7, Tyr15, Val16 and Ile25. The total stabilizing energetics at t = 0 ns and 300 ns frames for the cyclotide—Aβ_1-42_ fibril complex is detailed in Table 2.

**TABLE 2:** The total stabilizing energy at t = 0 ns and t = 300 ns for the disease relevant Y10A mutant Aβ_1-42_ fibril (PDB ID: 2NAO) interacting with cyclotide Cter-M (PDB ID: 2LAM).

Total stabilizing energy	0^th^ ns frame	300^th^ ns frame
Hydrogen Bond Energy (kJ/mol)	0	0
Electrostatic Energy (kJ/mol)	−6.34	−22.52
Van der Waals Energy (kJ/mol)	−159.43	−152.48
Total Stabilizing Energy (kJ/mol)	−165.77	−175.01
Number of interface residues	67	63
Normalized Energy per residue (kJ/mol)	−2.47	−2.78
No. of Hydrophobic Interactions	4	5
No. of van der Waals Pairs	6,558	6,161
No. of Salt Bridges	0	1
No. of Potential Favourable Electrostatic Interactions	3	5

### 3.4 Conformational changes in WT and mutant Aβ structures

**FIGURE 4:**
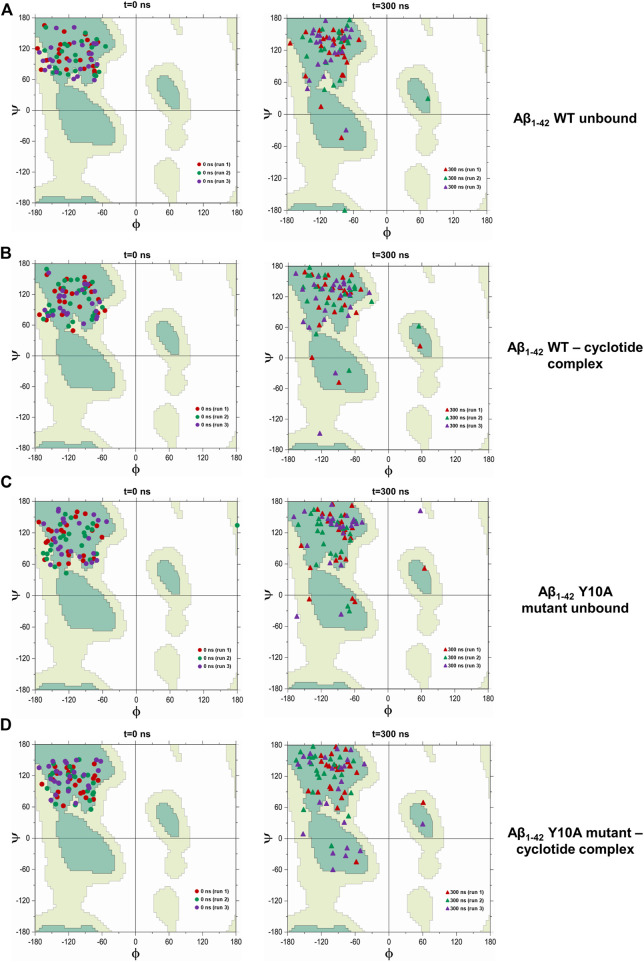
Scatter plot in φ–ψ space for residues 2–6 (chains A-C) and 39–41 (chains D-F) in the cyclotide binding pocket for **(A)** unbound wild-type Aβ_1-42_, **(B)** wild-type Aβ_1-42_—cyclotide complex, **(C)** Aβ_1-42_ Y10A mutant unbound and **(D)** Aβ_1-42_ Y10A mutant—cyclotide complex, at the beginning (0^th^ ns) and end (300^th^ ns) of simulation trajectories. Fully allowed and favourable regions in the φ–ψ space are shown in the background of Ramachandran Map that was redrawn by the authors of the current study, using a javascript provided by Peter N. Robinson (http://compbio.charite.de/contao/index.php/ramachandran.html).

Our analysis of secondary structural variations during the 300 ns MD simulations indicate that extended β-sheet conformations were stable throughout the trajectory in the unbound wild-type Aβ_1-42_ fibril. However, in the cyclotide-Aβ_1-42_ bound trajectory, both in the WT and Y10A mutant, the binding pocket comprising of residues 2-6 of chains A-C. Residues 39–41 of chains D-F undergo significant structural changes soon after 50 ns. Further, the extended parallel β-sheet conformation, that is a known structural hallmark of Aβ molecules, gets lost. Figure 4 highlights the dihedral angles (φ-ѱ) distribution of residues 2–6 (chains A-C) and residues 39–41 (chains D-F) on the Ramachandran Map for 4 Aβ conformations i.e., WT Aβ_1-42_ unbound, WT Aβ_1-42_—cyclotide complex, Aβ_1-42_ Y10A mutant unbound and Aβ_1-42_ Y10A mutant—cyclotide complex, at the beginning (0^th^ ns) and end (300^th^ ns) of simulation trajectories. The deviation from ideal φ-ѱ values of the β-sheet to ɑ_R_ region was observed in all the structures except in the unbound wild-type form of Aβ_1-42_. This observation indicates that, while the Y10A mutation causes some disruption to the β-sheet integrity of the binding pocket in Aβ_1-42_, even more disruptive is the cyclotide binding. It is as though the Aβ_1-42_ fibril is under a “double threat and attack” from not just an inherent mutation at a key structural site, but also from cyclotide inhibiting the fibril aggregation.

### 3.5 Structure-based thermodynamic properties in WT and mutant Aβ structures

**FIGURE 5:**
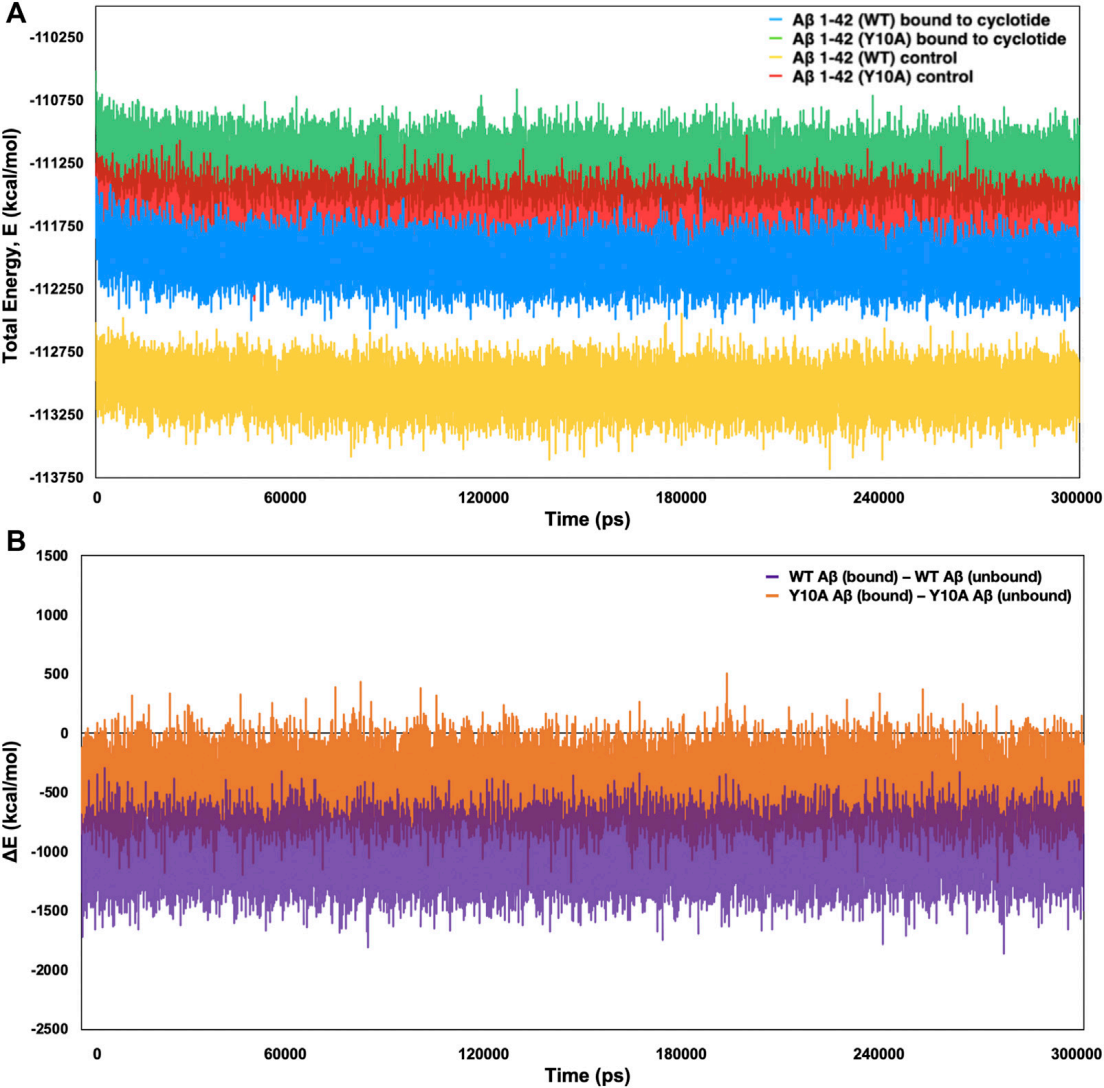
Total (E) and difference (Δ) in energy terms, ΔE = E _bound_—E _unbound_ of the system as a function of MD simulation time (300 ns) for wild-type and Tyr10Ala mutant Aβ_1-42_ (PDB ID: 2NAO) in complex with cyclotide (PDB ID: 2LAM) and their corresponding control (unbound) trajectories. **(A)** The total energy and **(B)** ΔE terms for the different systems are plotted in different colours and mentioned in the corresponding legend. Both the thermodynamic parameters are plotted in the units of kcal mol^−1^ vs. time (ps).

To further confirm the thermodynamic instability in the Aβ structure upon cyclotide binding, the total energies were calculated for the representative structures of wild-type Aβ_1-42_ (PDB ID: 2NAO) and Y10A mutant form over the length of the simulation within the Desmond package. The energy value was collected at 10 ps intervals from the 300 ns MD simulation trajectory, resulting in a total of 30,000 data points each for WT Aβ_1-42_ and Y10A mutant structure of Aβ_1-42_ fibril, in cyclotide bound and unbound forms. Figure 5A highlights the total energy (kcal/mol) of the Aβ_1-42_ and the Y10A mutant structures as a function of simulation time (300 ns). The analysis revealed an average total energy of −112,034.3 kcal/mol for WT Aβ_1-42_ bound to cyclotide (please see Table 3 for precise values). For the corresponding control trajectory of WT Aβ_1-42_, the average total energy of −113,048.8 kcal/mol was noted. In the case of the mutant complex, the analysis showed an average total energy of −111,248.3 kcal/mol for cyclotide bound to Y10A mutant form of Aβ_1-42_, and an average total energy of −111,668.4 kcal/mol for its corresponding control trajectory i.e., unbound mutant Aβ_1-42_. It is evident that the total energy of the Aβ fibril is higher in unbound forms of Aβ_1-42_ and decreases upon cyclotide binding, both in the WT and Y10A mutant forms (Figure 5A; Table 3). To understand if Y10A mutation stabilizes or destabilizes the cyclotide binding event in Aβ_1-42_, we calculated the difference in energy terms (ΔE) between the total energy of Aβ_1-42_ bound to cyclotide and corresponding unbound form, and compared the difference with that of the WT Aβ_1-42_. It is evident from Figure 5B that *in silico* mutation of Tyrosine 10 to Alanine reduced the affinity of the cyclotide with Aβ_1-42_, thereby highlighting the importance of this residue in efficient ligand recognition. In future studies, it will be worthwhile to assess the effects of Tyrosine 10 mutation *in vitro* to confirm its role in ligand recognition and ligand-Aβ interactions. The energy trends estimated in the present study not only demonstrate that the simulation runs have equilibrated, and the fluctuations were stabilized, but also show that they are reliable estimates of thermodynamic instability in Aβ_1-42_ fibril when cyclotide binding occurs.

**TABLE 3:** Average total energy and ΔE = E _bound_—E _unbound_, of the WT and Y10A forms of Aβ_1-42_ complexes bound to cyclotide and their corresponding control (unbound) trajectories across the 300 ns simulation time.

Extracted MD properties	WT Aβ_1-42_ fibril bound to cyclotide	WT Aβ_1-42_ fibril unbound	Y10A mutant Aβ_1-42_ bound to cyclotide	Y10A mutant Aβ_1-42_ unbound
	Avg. ± Std. Dev.	Avg. ± Std. Dev.	Avg. ± Std. Dev.	Avg. ± Std. Dev.
Total Energy, E (kcal/mol)	−112034.286 ± 141.646	−113048.818 ± 139.697	−111248.254 ± 144.482	−111668.352 ± 145.094
Difference in Total Energy, ΔE (kcal/mol)	−1,014.528 ± 194.386		−420.109 ± 199.832	

## 4 Discussion and conclusion

Cyclotides have displayed immense potential as peptide therapeutics for drug development for the treatment of diseases affecting the central nervous system and are now on their way from preclinical studies to clinical trials for blocking the progression of multiple sclerosis (Thell et al., 2016). More recently, their anti-neurodegenerative properties were studied in *P. solitudinum*, where they acted as inhibitors of the human prolyl oligopeptidase (POP), a promising target for the treatment of cognitive deficits in several psychiatric and neurodegenerative diseases (Hellinger et al., 2015). Highly constrained cyclic peptides have gained special attention for the modulation of protein-protein interactions. SFTI-1 (sunflower trypsin inhibitor-1) and kB1 (kalata B1) showed inhibitory activity against hexapeptide AcVQIVYK-NH2 (AcPHF6) involved in tau fibril formation, highlighting their appeal as candidates for inhibition of fibril formation (Wang et al., 2016). Stronger inhibition of the AcPHF6 fibril formation was observed when bound to kB1 than SFTI-1, another naturally occurring, disulfide-rich cyclic peptide, suggesting that the sequence and structure of kB1 makes it better at disrupting the fibrils. Furthermore, a recently published work from our group describes the anti-Aβ *in vivo* activity of cyclotides extracted from *C. ternatea* in three transgenic *C. elegans* strains that exhibit pathological behaviours associated with Aβ (Kalmankar et al., 2021) and thereby offering a novel pharmacophore lead against neurodegenerative diseases, particularly against β-amyloid fibril formation. In order to obtain a structural understanding of how the presence of cyclotides would reduce the aggregation of Aβ peptides in the transgenic nematodes, we modelled the interactions and performed MD simulations and the results are described in the detail in the present study.

*In silico* tools such as molecular docking and MD simulations allow one to know the affinity and interaction between the Aβ structures and its inhibitor scaffolds. Aβ is an extracellular protein and in its monomeric form in the membrane adopts an α-helical structure. There is a conformational transition into β-sheet rich structures in the process of aggregation (Xu et al., 2005). In this work, we evaluated the mode and stability of inter-molecular interactions between cyclotide and diverse models of Aβ. The results of a comprehensive structural analyses presented here shows that the cyclotide conformation and interactions with Aβ fibrils can be reinforced by hydrogen bonding, hydrophobic and long-range electrostatic interactions between key residues of Aβ peptide and cyclotides. We show a representative complex of disease-relevant Aβ_1-42_ fibril (PDB ID: 2NAO) and cyclotide Cter-M (PDB ID: 2LAM) to highlight the strong interactions between the two molecules. In order to analyse the structural consequences of cyclotide binding upon mutation in the Aβ, we implemented *in silico* mutagenesis at position 10, converting Tyr to Ala, in the disease-relevant Aβ_1-42_ fibril (PDB ID: 2NAO), and performed 300 ns MD simulations for the mutant forms of Aβ_1-42_. It was clearly evident that the protein-peptide pairs display several non-covalent interactions throughout the simulation period both in the WT Aβ_1-42_—cyclotide complex and the mutant Aβ_1-42_—cyclotide complex. The persistence of high number of intermolecular hydrogen bonds and strong non-covalent interactions throughout the trajectory highlights the stable binding of cyclotide in the exposed pockets of amyloid fibril. Moreover, cyclotide disrupts the inter-chain hydrogen bonds and salt bridges in Aβ which are crucial for the structural integrity and shape of the fibril. The important contribution from Tyr 10 in the binding pocket towards cyclotide binding and stability of Aβ was reported. Mutation of Y10A clearly led to a weaker binding affinity between the cyclotide–Aβ complex and these results highlight the necessity of tyrosine at position 10 for the stability of the Aβ aggregate. For future mutagenesis studies, apart from Tyr10, positions 4 and 13 could also be considered. Position 4 is conserved by a critical aromatic phenylalanine residue, contributing π-electrons not only important for π-π stacking in the self-assembly of amyloid fibrils but also for inhibitor interactions (Gazit et al., 2002; Adler-Abramovich et al., 2012). Position 13 contains a conserved histidine residue, and if mutated to alanine can have destabilization effects on the fibrillar assembly due to the protonation states of histidines and associated changes in pH levels (Brännström et al., 2017). Figure 2 clearly highlights the importance of these two N-terminal sites for cyclotide binding and destabilization, and can be potential sites for point-mutations.

In conclusion, the results of our extensive molecular docking and MD simulations efforts have enabled us to predict the mode of interaction between cyclotides and different physiological forms of Aβ structures. We have described how cyclotides bind tightly to the Aβ chains *via* strong hydrogen bonds, hydrophobic, electrostatic and π-π interactions and thereby inhibit Aβ aggregation process. Due to their immense potential in peptide therapeutics, cyclotides can be regarded as a new class of cyclic peptide Aβ inhibitors.

## Data Availability

The original contributions presented in the study are included in the article/Supplementary Material, further inquiries can be directed to the corresponding author.
