# Supplementary material for: Effects of a plant cyclotide on conformational dynamics and destabilization of β-amyloid fibrils through molecular dynamics simulations
Source: Front Mol Biosci. 2022 Sep 30;9:986704. doi: 10.3389/fmolb.2022.986704 (PMC9561823; doi:10.3389/fmolb.2022.986704)
Supplement: Supplementary file 1 [file DataSheet1.PDF]

Supplementary Material

Supplementary Table S1. Results of PPCheck webserver for predicting normalized energy per residue for FRODOCK docked models. Best docking pose for each protein-peptide complex is highlighted in orange.

a) 1IYT-2LAM

Decoy-ID	Hydrogen Bond Energy (kJ/mol)	Electrostatic Energy (kJ/mol)	van der Waals Energy (kJ/mol)	Total Energy (kJ/mol)	Number of Interface Residues	Normalized Energy per Residue (kJ/mol)
pose2	0.00	-2.58	-99.83	-102.41	45	-2.28
pose4	0.00	0.00	-101.76	-101.76	49	-2.08
pose6	0.00	-2.20	-76.59	-78.79	45	-1.75
pose5	0.00	1.06	-22.65	-21.58	51	-0.42
pose8	0.00	0.00	-20.65	-20.65	52	-0.40
pose3	0.00	0.00	7.13	7.13	50	0.14
pose10	0.00	0.00	36.74	36.74	51	0.72
pose1	-8.40	0.00	86.88	78.48	46	1.71
pose7	0.00	0.00	261.67	261.67	49	5.34
pose9	0.00	0.00	365.36	365.36	51	7.16

b) 2BEG-2LAM

Decoy-ID	Hydrogen Bond Energy (kJ/mol)	Electrostatic Energy (kJ/mol)	van der Waals Energy (kJ/mol)	Total Energy (kJ/mol)	Number of Interface Residues	Normalized Energy per Residue (kJ/mol)
pose3	0.00	0.00	-91.52	-91.52	49	-1.87
pose4	0.00	0.00	-79.78	-79.78	53	-1.51
pose10	0.00	0.00	-12.82	-12.82	51	-0.25
pose9	0.00	0.00	74.80	74.80	61	1.23
pose7	-2.88	0.00	77.83	74.94	42	1.78
pose5	0.00	0.00	114.80	114.80	60	1.91
pose8	0.00	0.00	174.19	174.19	43	4.05
pose1	0.00	0.00	387.97	387.97	47	8.25
pose2	-19.84	0.00	543.28	523.44	43	12.17
pose6	0.00	0.00	819.29	819.29	67	12.23

c) 2MXU-2LAM

Decoy-ID	Hydrogen Bond Energy (kJ/mol)	Electrostatic Energy (kJ/mol)	van der Waals Energy (kJ/mol)	Total Energy (kJ/mol)	Number of Interface Residues	Normalized Energy per Residue (kJ/mol)
pose7	0.00	0.00	-70.81	-70.81	84	-0.84
pose6	-4.13	0.00	-17.44	-21.57	78	-0.28
pose1	-16.55	0.00	139.07	122.51	89	1.38
pose9	0.00	0.00	158.42	158.42	74	2.14
pose8	0.00	0.00	170.22	170.22	76	2.24
pose4	0.00	0.00	302.98	302.98	74	4.09
pose3	-7.25	0.00	362.80	355.55	84	4.23
pose10	0.00	0.00	463.90	463.90	81	5.73
pose5	0.00	0.00	616.12	616.12	94	6.55
pose2	-12.92	0.00	1034.95	1022.03	98	10.43

d) 2NAO-2LAM

Decoy-ID	Hydrogen Bond Energy (kJ/mol)	Electrostatic Energy (kJ/mol)	van der Waals Energy (kJ/mol)	Total Energy (kJ/mol)	Number of Interface Residues	Normalized Energy per Residue (kJ/mol)
pose10	-4.17	0.00	-95.50	-99.67	65.00	-1.53
pose5	-4.78	0.00	-15.15	-19.94	72.00	-0.28
pose8	-14.51	4.94	146.56	136.99	73.00	1.88
pose7	0.00	0.00	135.39	135.39	68.00	1.99
pose1	0.00	0.00	144.70	144.70	63.00	2.30
pose6	-4.27	0.00	254.73	250.46	70.00	3.58
pose2	-18.43	12.34	337.50	331.40	84.00	3.95
pose4	0.00	0.00	324.05	324.05	76.00	4.26
pose3	-7.91	0.00	382.54	374.63	71.00	5.28
pose9	-8.57	0.00	1144.61	1136.04	82.00	13.85

(a) Secondary structure MD timeline plot of 2NAO (A$\beta_{1\text{-}42}$) unbound

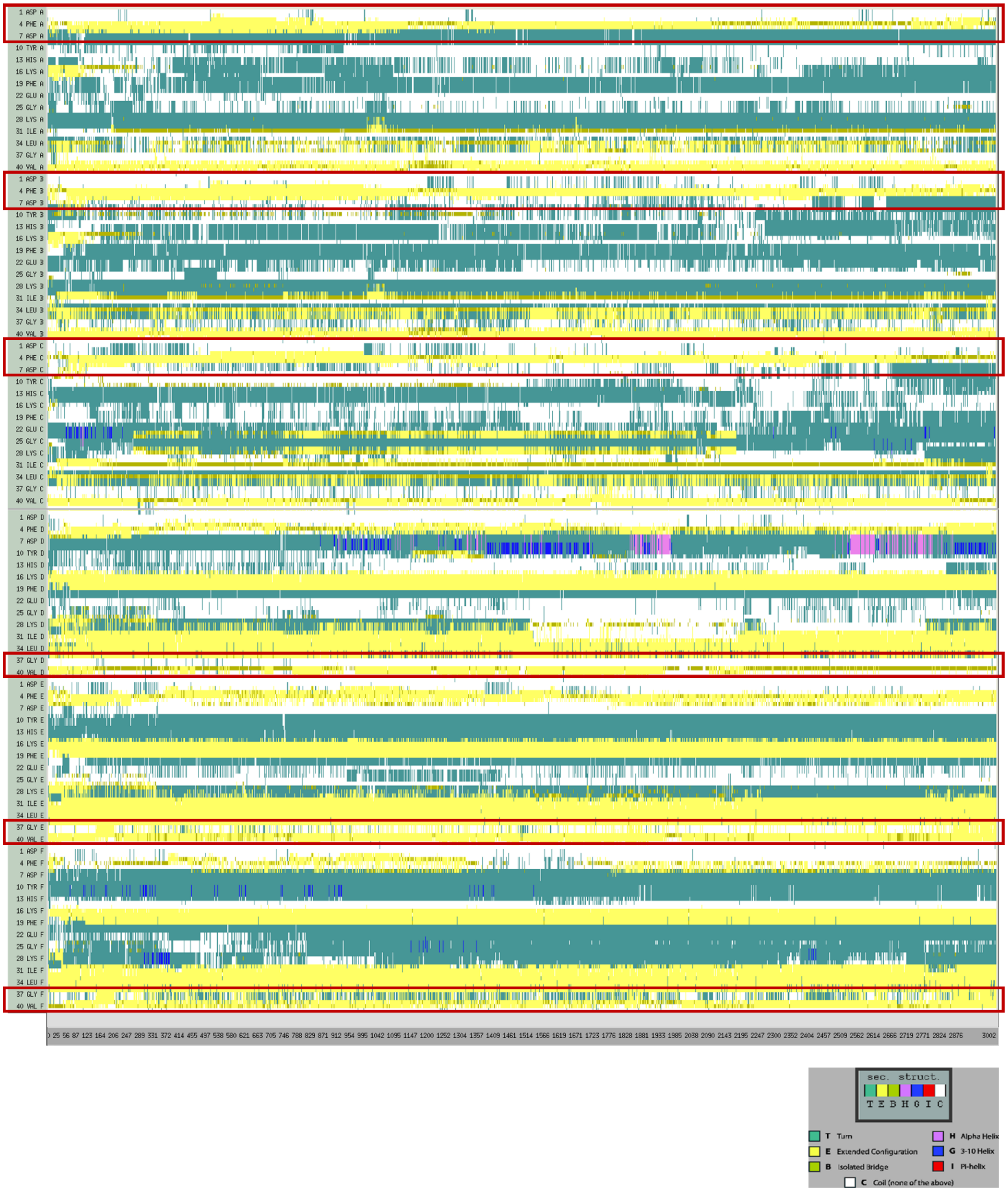

(b) Secondary structure MD timeline plot of 2NAO (Aβ$_{1-42}$) – 2LAM (Cter M) bound

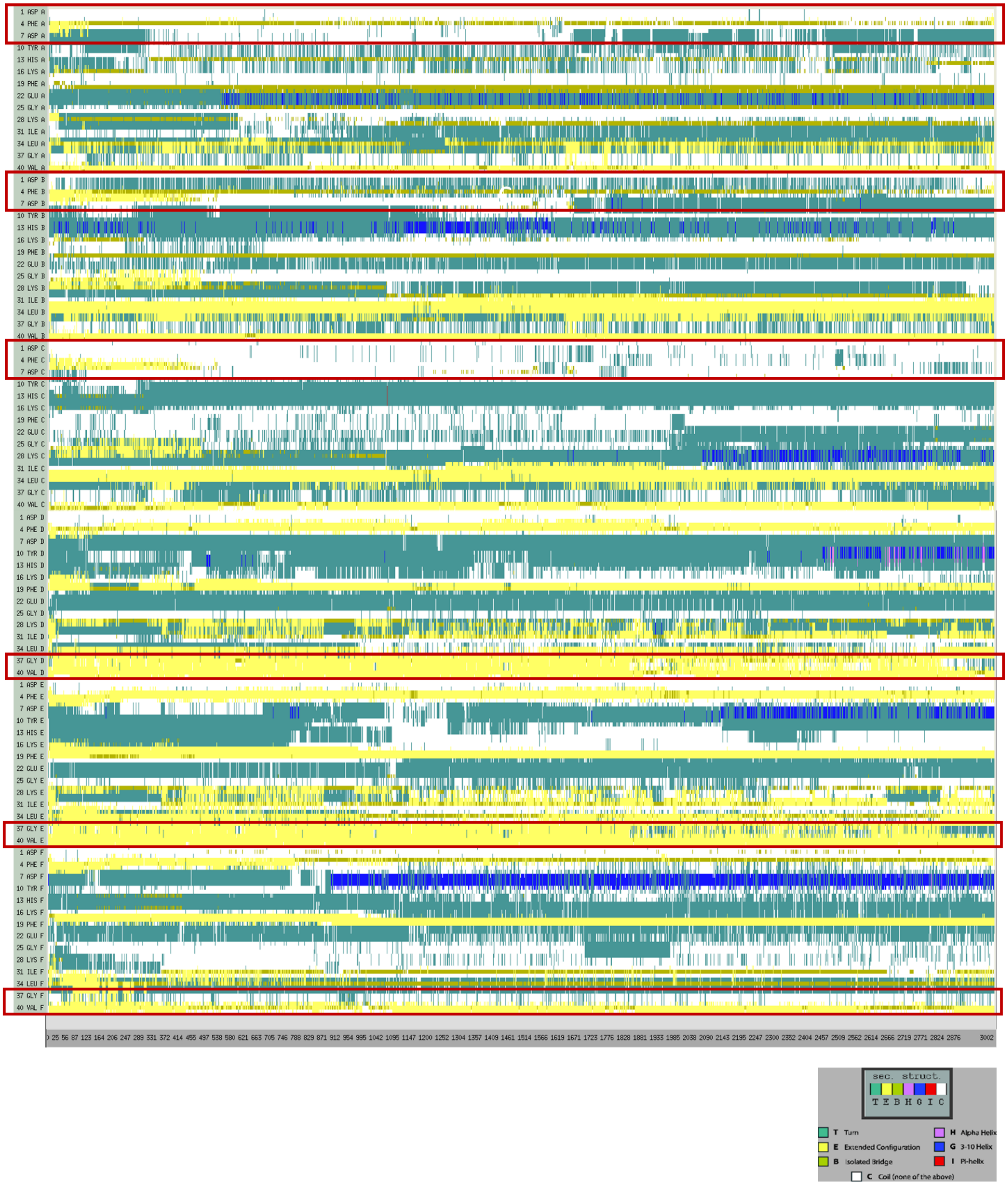

Supplementary Figure S1. Secondary structure timeline analysis during 300 ns MD simulation of (a) Aβ$_{1-42}$ fibril (PDB ID: 2NAO) (a) unbound and (b) bound to cyclotide Cter-M.

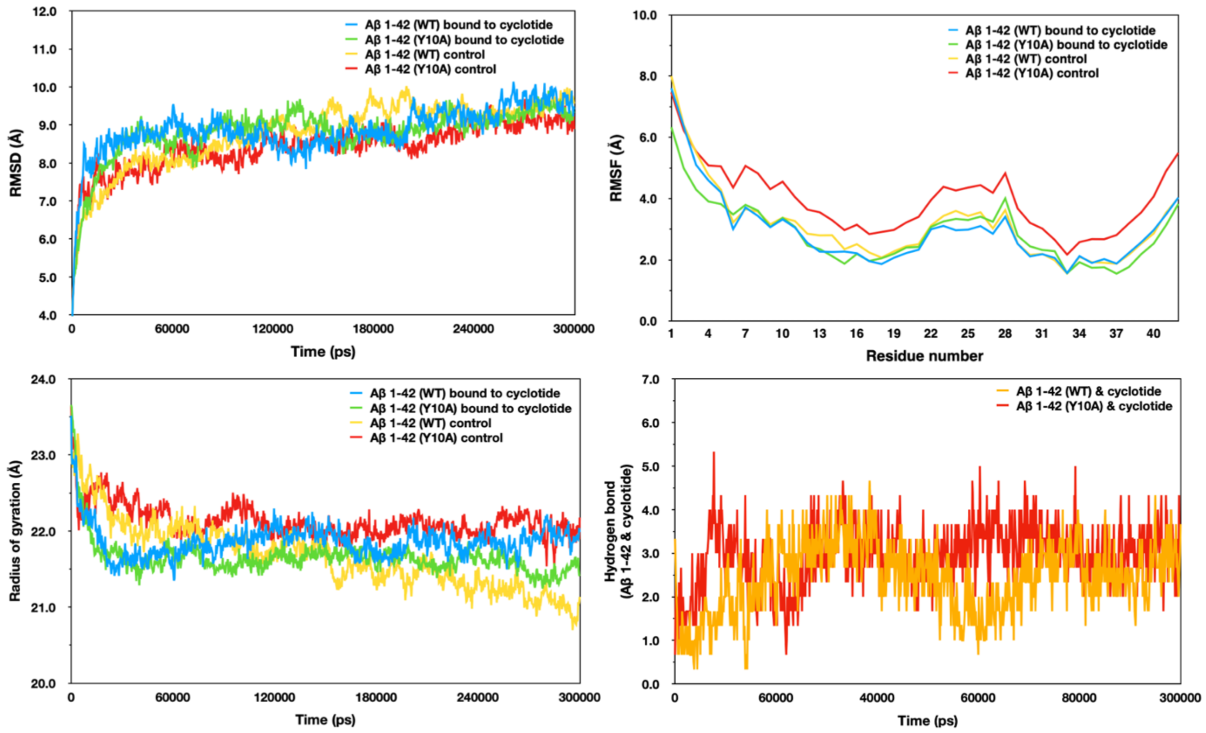

Supplementary Figure S2. Plots of triplicate 300 ns MD simulations for cyclotide (PDB ID: 2LAM) and wildtype and mutant A$\beta_{1\text{-}42}$ fibrils (PDB ID: 2NAO) complex, averaged over triplicate simulations. RMSD (top left panel), RMSF (top right panel), Radius of gyration (bottom left panel), number of hydrogen bonds (bottom right panel).

Supplementary Table S2. Analysis of protein-protein interactions at t=0 ns (top) and t=300ns (bottom) frames between cyclotide Cter-M (PDB ID: 2LAM) and wildtype A$\beta_{1\text{-}42}$ fibril (PDB ID: 2NAO).

Interactions	[a]Maestro	[a]PPCheck	2NAO				2LAM				[b]Type of Bond	Distance Å
			Residue-1				Residue-2					
			Res No.	Res Name	Chain	Atom Name	Res No.	Res Name	Chain	Atom Name		
**t=0 ns**												
Hydrogen Bond	✓	-	13	HIS	C	NE2	7	GLU	Z	N	SB	3.2
	✓	-	13	HIS	B	ND1	3	PRO	Z	O	SB	3.7
	✓	-	14	HIS	B	NE2	6	GLU	Z	N	SB	3.5
	✓	✓	38	GLY	E	O	15	TYR	Z	OH	BS	2.7
	-	✓	39	VAL	F	N	15	TYR	Z	OH	BS	2.8
	-	✓	38	GLY	F	N	15	TYR	Z	OH	BS	2.9
	✓	-	4	PHE	C	CZ	29	ASN	Z	O	SB	3.8

Aromatic Hydrogen Bond	✓	-	6	HIS	C	CE1	1	GLY	Z	O	SB	4.4
	✓	-	13	HIS	C	CE1	26	CYS	Z	O	SB	3.2
Hydrophobic Interactions	-	✓	10	TYR	C	CB	25	ILE	Z	CB	SS	5.0
	-	✓	40	VAL	D	CB	16	VAL	Z	CB	SS	6.9
	-	✓	40	VAL	E	CB	15	TYR	Z	CB	SS	6.5
	-	✓	40	VAL	E	CB	16	VAL	Z	CB	SS	6.6
	-	✓	40	VAL	F	CB	15	TYR	Z	CB	SS	5.3
Electrostatic Interactions	-	✓	13	HIS	C	CB	7	GLU	Z	CB	SS	7.9
	-	✓	14	HIS	C	CB	7	GLU	Z	CB	SS	7.6
**t=300 ns**												
Hydrogen Bond	✓	✓	6	HIS	C	N	1	GLY	Z	O	BB	3.0
	✓	✓	38	GLY	D	N	6	GLY	Z	O	BB	2.7
	✓	-	38	GLY	F	O	15	TYR	Z	OH	SB	3.3
Aromatic Hydrogen Bond	✓	-	4	PHE	C	CE2	1	GLY	Z	O	SB	3.4
Hydrophobic Interactions	-	✓	10	TYR	C	CB	25	ILE	Z	CB	SS	5.6
	-	✓	40	VAL	D	CB	15	TYR	Z	CB	SS	5.0
	-	✓	40	VAL	D	CB	16	VAL	Z	CB	SS	5.5
	-	✓	40	VAL	E	CB	15	TYR	Z	CB	SS	4.8
	-	✓	39	VAL	F	CB	11	LEU	Z	CB	SS	7.0
Electrostatic Interactions	-	✓	13	HIS	A	CB	7	GLU	Z	CB	SS	9.3

[a] Protein-protein interaction identified from Maestro and/or PPCheck is highlighted with ✓.
[b] "SS" represents sidechain-sidechain interaction, "SB" represents sidechain-backbone interaction, and "BB" represents backbone-backbone mode of interaction between the two interacting amino acids.

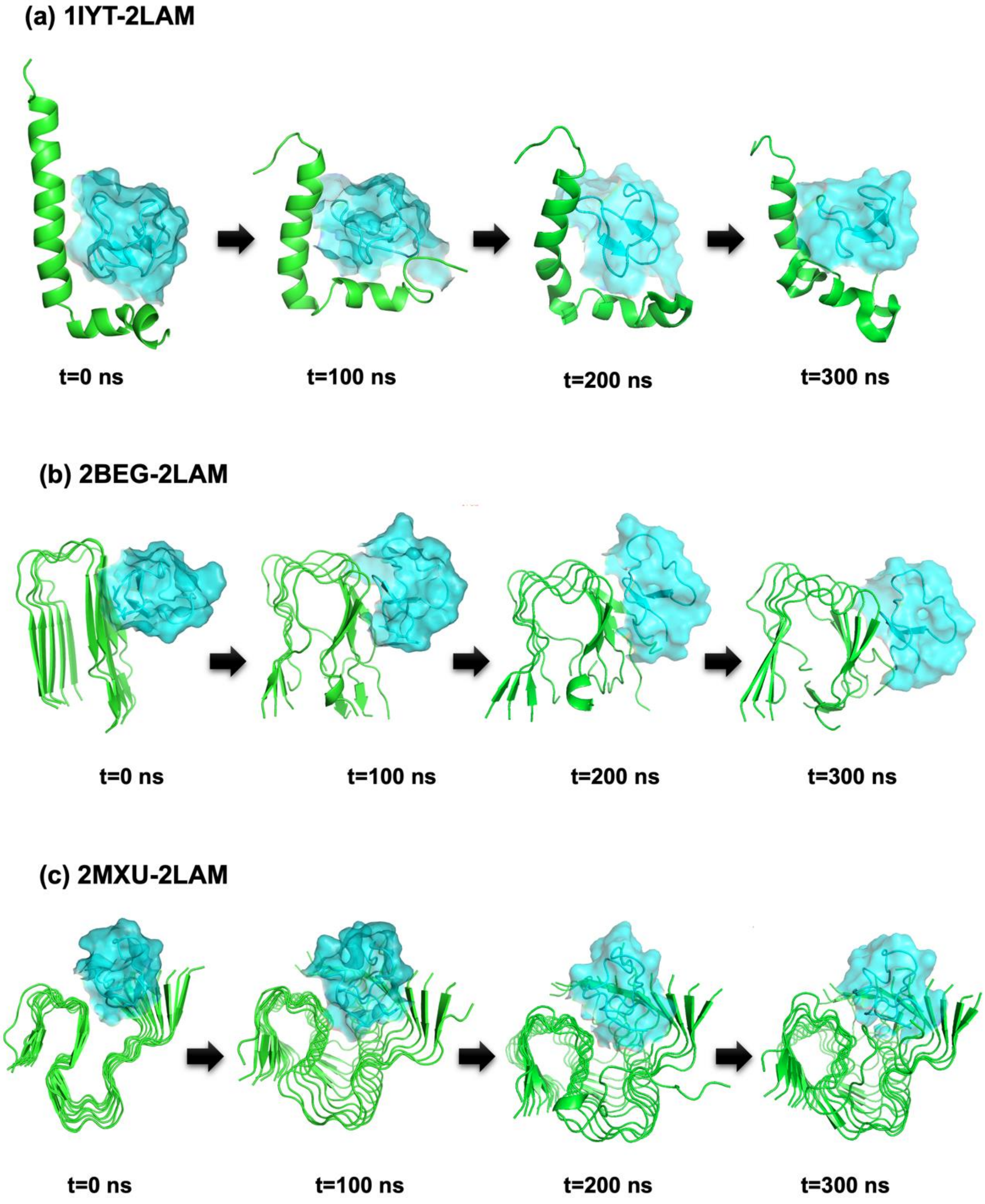

Supplementary Figure S3. Snapshots of MD simulation of cyclotide (PDB ID: 2LAM; cyan surface representation) and (A) Aβ_{1-42} monomer (PDB ID: 1IYT, green cartoon representation), (B) Aβ_{17-42} U-shaped pentamer (PDB ID: 2BEG, green cartoon representation) and (C) Aβ_{11-42} S-shaped model (PDB ID: 2MXU, green cartoon representation) complexes at different time points along the simulation period.

(a) 1IYT-2LAM

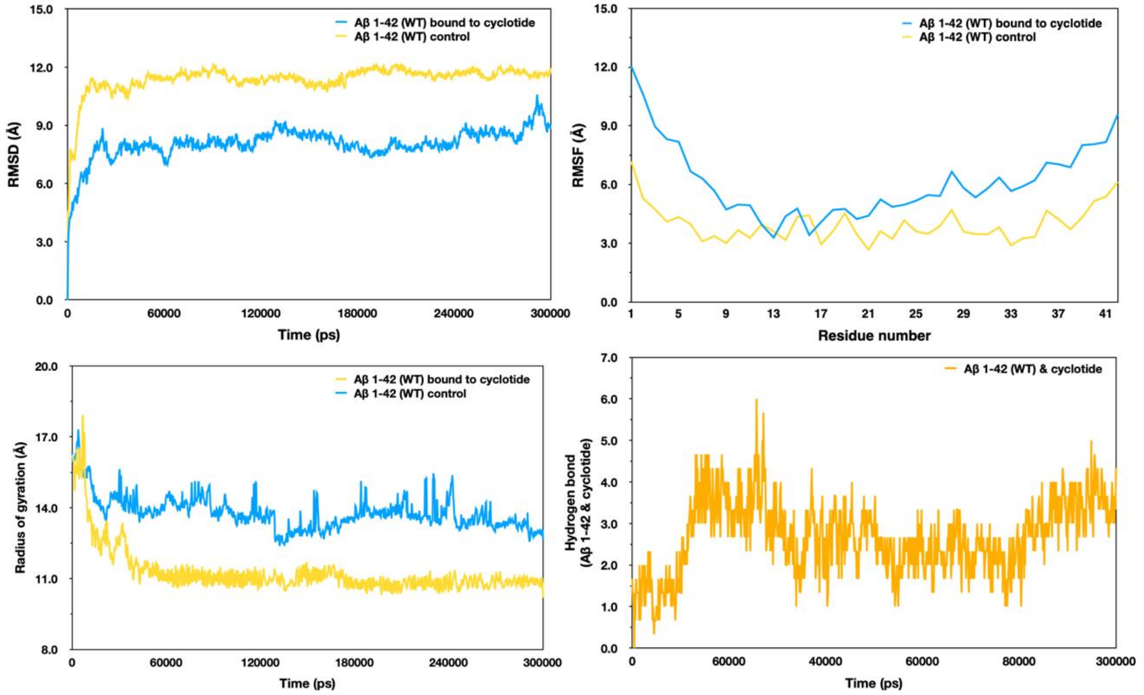

(b) 2BEG-2LAM

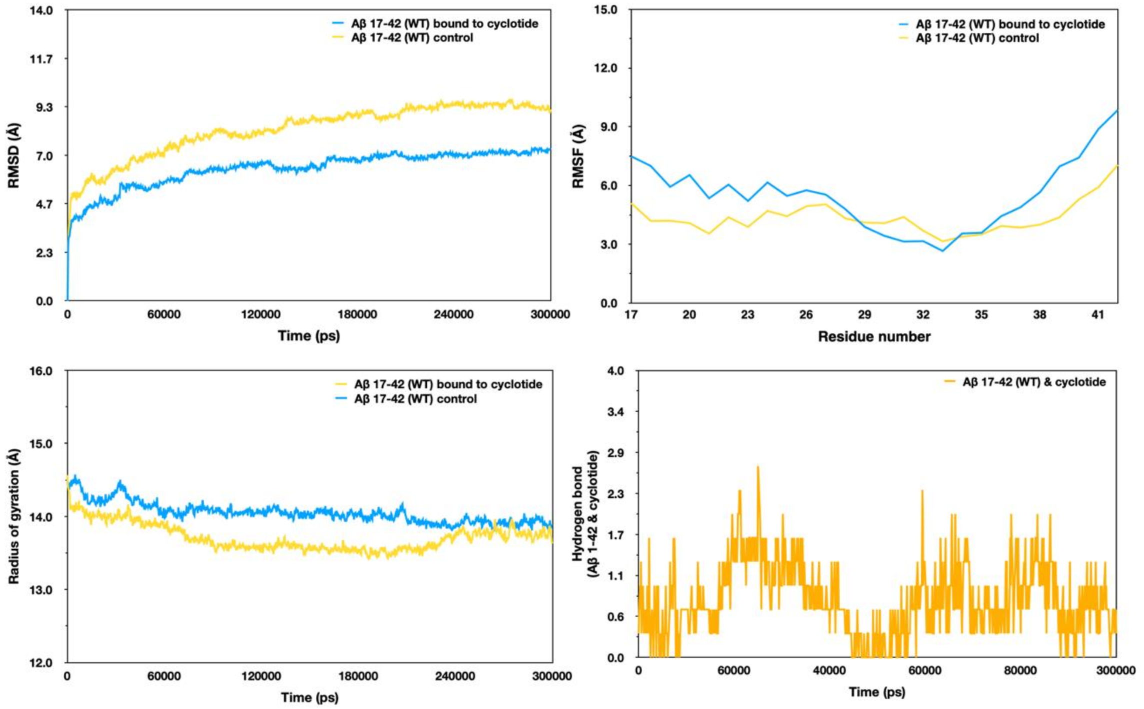

(c) 2MXU-2LAM

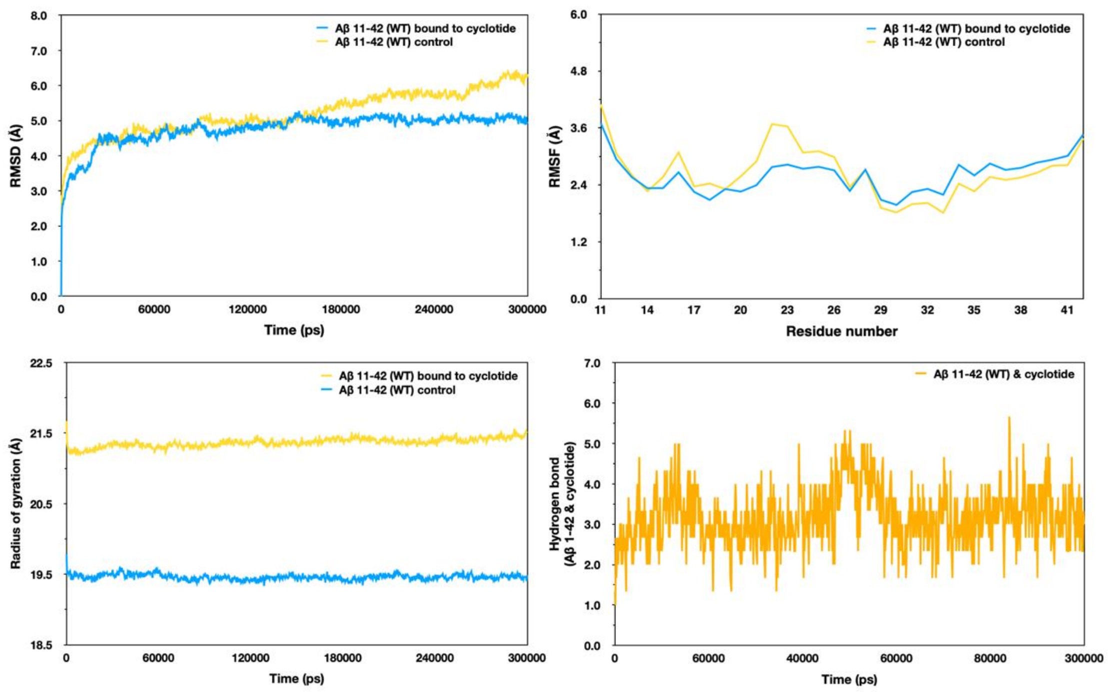

Supplementary Figure S4. Plots of 300ns simulations showing RMSD (top left panel), RMSF (top right panel), Radius of gyration (bottom left panel) and number of hydrogen bonds (bottom right panel) for complexes between cyclotide (PDB ID: 2LAM) and (A) Aβ_{1-42} monomer (PDB ID: 1IYT), (B) Aβ_{17-42} U-shaped pentamer (PDB ID: 2BEG) and (C) Aβ_{11-42} S-shaped model (PDB ID: 2MXU).

Supplementary Table S3. The total stabilizing energy at t=0 ns and t=300 ns for three other cyclotide - Aβ complexes.

**Total Stabilizing Energy**	**1IYT-2LAM**		**2BEG-2LAM**		**2MXU-2LAM**	
	**t=0 ns**	**t=300 ns**	**t=0 ns**	**t=300 ns**	**t=0 ns**	**t=300 ns**
Hydrogen Bond Energy (kJ/mol)	0.00	-25.40	0.00	0.00	-8.83	-6.63
Electrostatic Energy (kJ/mol)	0.00	-23.30	0.00	0.00	0.00	0.00
Van der Waals Energy (kJ/mol)	-97.70	-119.59	-123.14	-141.98	-180.95	-208.46
Total Stabilizing Energy (kJ/mol)	-97.70	-168.29	-123.14	-141.98	-189.77	-215.08
Number of interface residues	43	48	52	54	87	93
Normalized Energy per residue (kJ/mol)	-2.27	-3.51	-2.37	-2.63	-2.18	-2.31
No. of Hydrophobic Interactions	0	1	2	0	1	3
No. of van der Waals Pairs	3791	4532	5534	6360	8970	9920
No. of Salt Bridges	0	1	0	0	0	0
No. of Potential Favourable Electrostatic Interactions	0	2	0	0	0	0

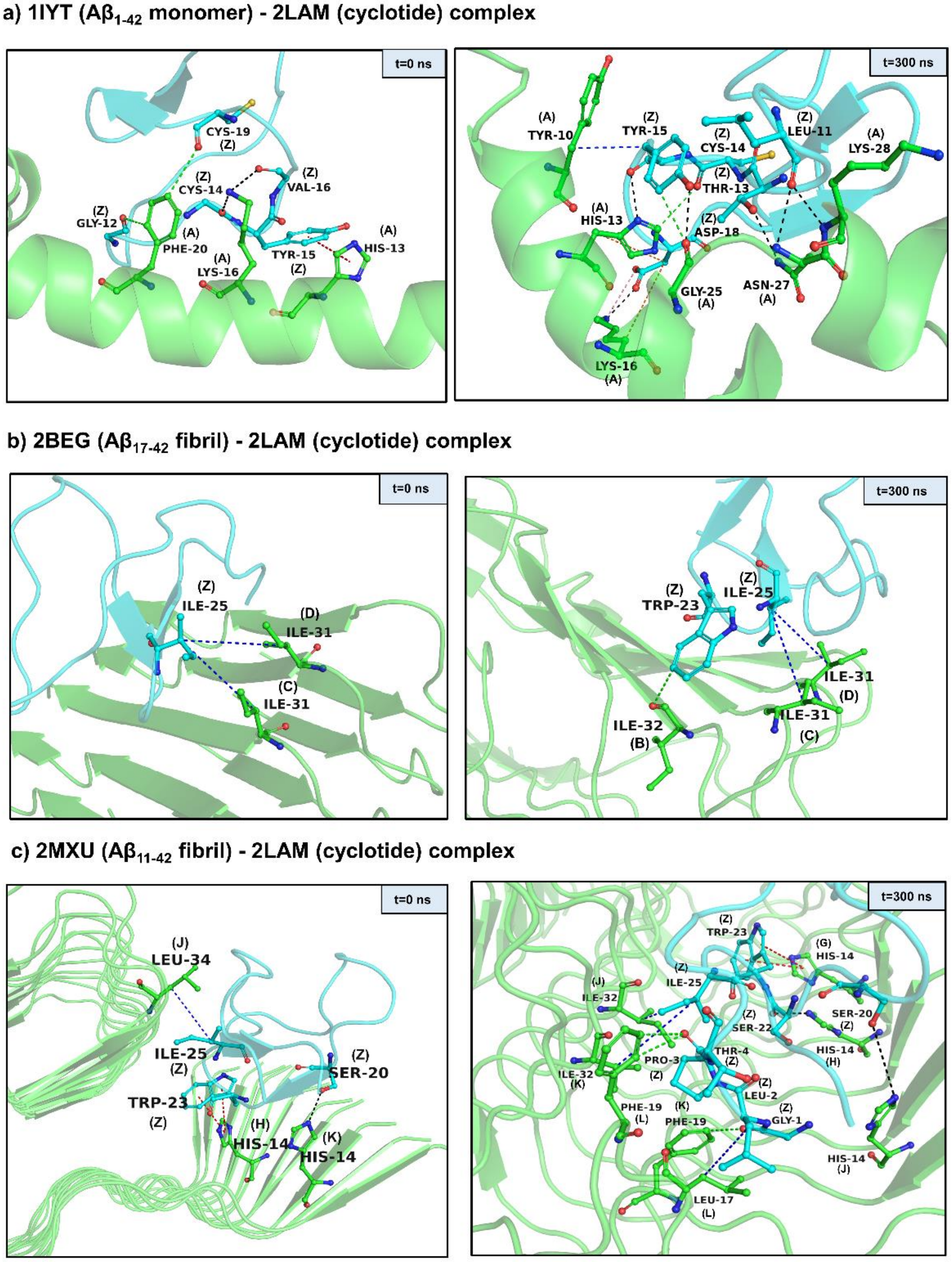

Supplementary Figure S5. Molecular interactions between cyclotide (PDB ID: 2LAM; chain Z; cyan cartoon representation) and (A) A$\beta_{1\text{-}42}$ monomer (PDB ID: 1IYT; chain A; green cartoon representation), (B) A$\beta_{17\text{-}42}$ U-shaped pentamer (PDB ID: 2BEG; chain A-E; green cartoon representation) and (C) A$\beta_{11\text{-}42}$ S-shaped model (PDB ID: 2MXU; chain A-L; green cartoon representation), at the beginning (0th ns snapshot) and end (300th ns snapshot) of the simulation period. Colour scheme for interactions used: classical hydrogen bonds in black, aromatic hydrogen bonds in green, hydrophobic interactions in blue, electrostatic interactions in orange, salt-bridges in pink and π-π interactions in red.

Supplementary Table S4. Analysis of protein-protein interactions at t=0 ns (top) and t=300ns (bottom) frames between cyclotide (PDB ID: 2LAM) and Aβ_{1-42} monomer (PDB ID: 1IYT).

Interactions	[a]Maestro	[a]PPCheck	1IYT				2LAM				[b]Type of Bond	Distance Å
			Residue-1				Residue-2					
			Res No.	Res Name	Chain	Atom Name	Res No.	Res Name	Chain	Atom Name		
**t=0 ns**												
Hydrogen Bond	✓	-	16	LYS	A	NZ	14	CYS	Z	O	SB	3.3
	✓	-	16	LYS	A	NZ	16	VAL	Z	O	SB	2.8
Aromatic Hydrogen Bond	✓	-	20	PHE	A	CD2	12	GLY	Z	O	SB	3.6
	✓	-	20	PHE	A	CZ	19	CYS	Z	O	SB	3.5
π-π Interactions	✓	-	13	HIS	H	ring	15	TYR	Z	ring	SS	5.1
**t=300 ns**												
Hydrogen Bond	✓	-	13	HIS	A	ND1	15	TYR	Z	O	SB	2.7
	✓	-	25	GLY	A	O	15	TYR	Z	OH	BS	2.7
	-	✓	27	ASN	A	N	11	LEU	Z	O	BB	3.1
	✓	✓	27	ASN	A	N	13	THR	Z	OG1	BS	3.0
	✓	✓	28	LYS	A	N	11	LEU	Z	O	BB	3.0
	✓	✓	16	LYS	A	NZ	18	ASP	Z	OD1	SS	2.7
	✓	-	16	LYS	A	NZ	18	ASP	Z	OD2	SS	4.2
Aromatic Hydrogen Bond	✓	-	25	GLY	A	O	15	TYR	Z	CE2	BS	3.4
	✓	-	13	HIS	A	CE1	14	CYS	Z	O	SB	3.4
Hydrophobic Interactions	-	✓	10	TYR	A	CB	15	TYR	Z	CB	SS	4.2
Electrostatic Interactions	-	✓	13	HIS	A	CB	18	ASP	Z	CB	SS	7.3
	-	✓	16	LYS	A	CB	18	ASP	Z	CB	SS	7.6

[a] Protein-protein interaction identified from Maestro and/or PPCheck is highlighted with as ✓.
[b] "SS" represents sidechain-sidechain interaction, "SB" represents sidechain-backbone interaction, and "BB" represents backbone-backbone mode of interaction between the two interacting amino acids.

Supplementary Table S5. PPCheck results for protein-protein interactions at t=0 ns (top) and t=300ns (bottom) frames between cyclotide (PDB ID: 2LAM) and Aβ_{17-42} U-shaped pentamer (PDB ID: 2BEG).

Interactions	[a]Maestro	[a]PPCheck	2BEG				2LAM				[b]Type of Bond	Distance Å
			Residue-1				Residue-2					
			Res No.	Res Name	Chain	Atom Name	Res No.	Res Name	Chain	Atom Name		
**t=0 ns**												
Hydrophobic Interactions	-	✓	31	ILE	C	CB	25	ILE	Z	CB	SS	6.4
	-	✓	31	ILE	D	CB	25	ILE	Z	CB	SS	6.5

**t=300 ns**											
Aromatic Hydrogen Bond	✓	-	32	ILE	B	O	23	TRP	Z	CZ3	BS	3.3
Hydrophobic Interactions	-	✓	31	ILE	C	CB	25	ILE	Z	CB	SS	6.6
	-	✓	31	ILE	D	CB	25	ILE	Z	CB	SS	5.8

[a] Protein-protein interaction identified from Maestro and/or PPCheck is highlighted with as ✓.
[b] "SS" represents sidechain-sidechain interaction, "SB" represents sidechain-backbone interaction, and "BB" represents backbone-backbone mode of interaction between the two interacting amino acids.

Supplementary Table S6. PPCheck results for protein-protein interactions at t=0 ns (top) and t=300ns (bottom) frames between cyclotide (PDB ID: 2LAM) and Aβ_{11-42} S-shaped model (PDB ID: 2MXU).

**Interactions**	**[a]Maestro**	**[a]PPCheck**	**2MXU**				**2LAM**				**[b]Type of Bond**	**Distance Å**
			**Residue-1**				**Residue-2**					
			**Res No.**	**Res Name**	**Chain**	**Atom Name**	**Res No.**	**Res Name**	**Chain**	**Atom Name**		
**t=0 ns**												
Hydrogen Bond	✓	✓	14	HIS	K	NE2	20	SER	Z	OG	SS	3.1
Hydrophobic Interactions	-	✓	34	LEU	J	CB	25	ILE	Z	CB	SS	5.4
π-π Interactions	✓	-	14	HIS	H	ring	23	TRP	Z	ring 1	SS	4.9
	✓	-	14	HIS	H	ring	23	TRP	Z	ring 2	SS	4.5
**t=300 ns**												
Hydrogen Bond	-	✓	14	HIS	J	NE2	20	SER	Z	OG	SS	4.8
	✓	✓	14	HIS	H	NE2	22	SER	Z	O	SB	2.8
Aromatic Hydrogen Bond	✓	-	19	PHE	K	CZ	1	GLY	Z	O	SB	3.4
	✓	-	19	PHE	L	CD2	3	PRO	Z	O	SB	3.5
	✓	-	19	PHE	L	CE2	4	THR	Z	O	SB	3.6
Hydrophobic Interactions	-	✓	32	ILE	J	CB	25	ILE	Z	CB	SS	5.5
	-	✓	32	ILE	K	CB	25	ILE	Z	CB	SS	6.4
	-	✓	17	LEU	L	CB	2	LEU	Z	CB	SS	5.8
π-π Interactions	✓	-	14	HIS	G	ring 1	23	TRP	Z	ring	SS	5.1
	✓	-	14	HIS	G	ring 2	23	TRP	Z	ring	SS	4.5

[a] Protein-protein interaction identified from Maestro and/or PPCheck is highlighted with as ✓.
[b] "SS" represents sidechain-sidechain interaction, "SB" represents sidechain-backbone interaction, and "BB" represents backbone-backbone mode of interaction between the two interacting amino acids.

(a) Secondary structure MD timeline plot of 2NAO-Y10A ($A\beta_{1-42}$) mutant unbound

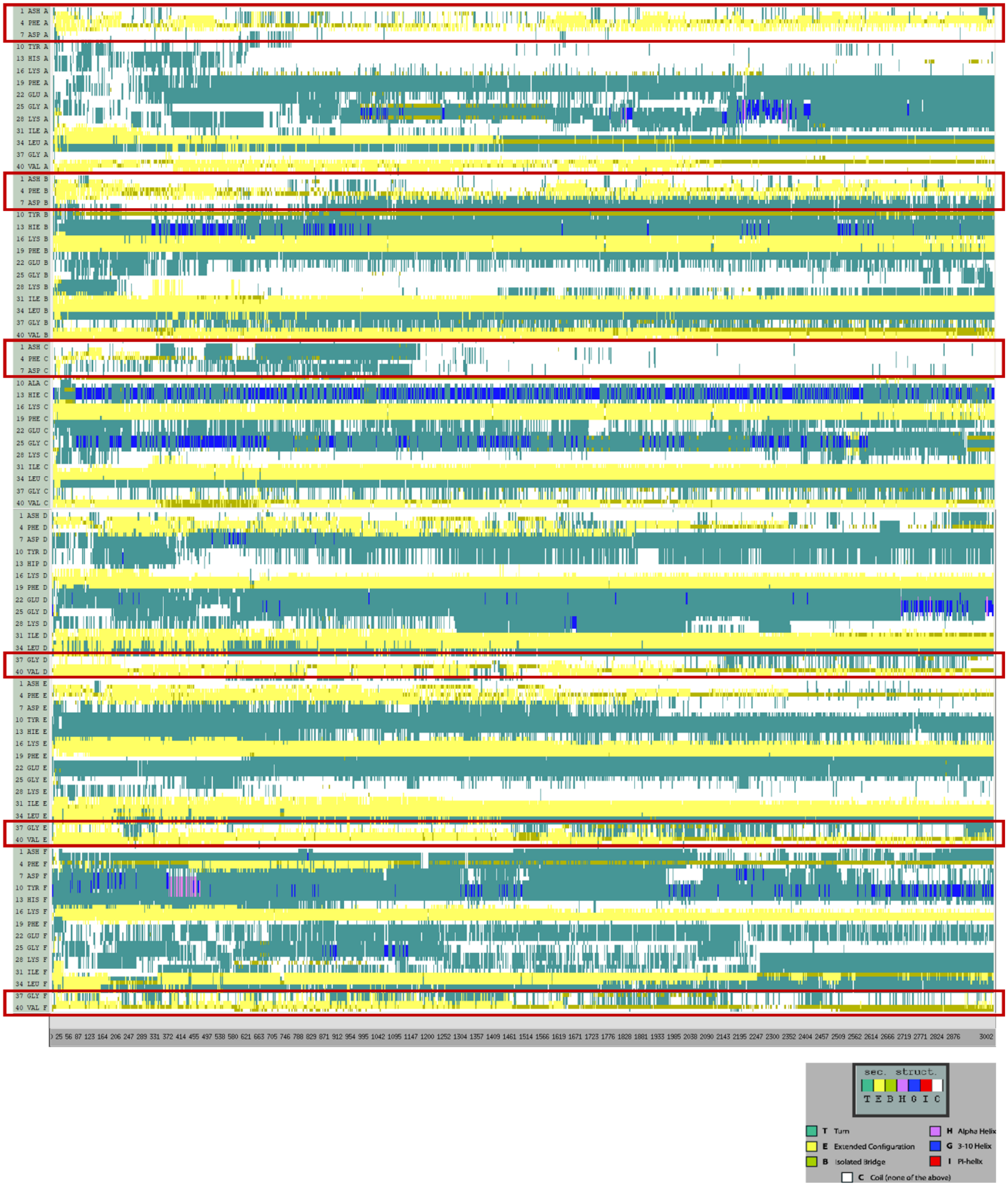

(b) Secondary structure MD timeline plot of 2NAO-Y10A (Aβ$_{1-42}$) mutant – 2LAM (Cter M) bound

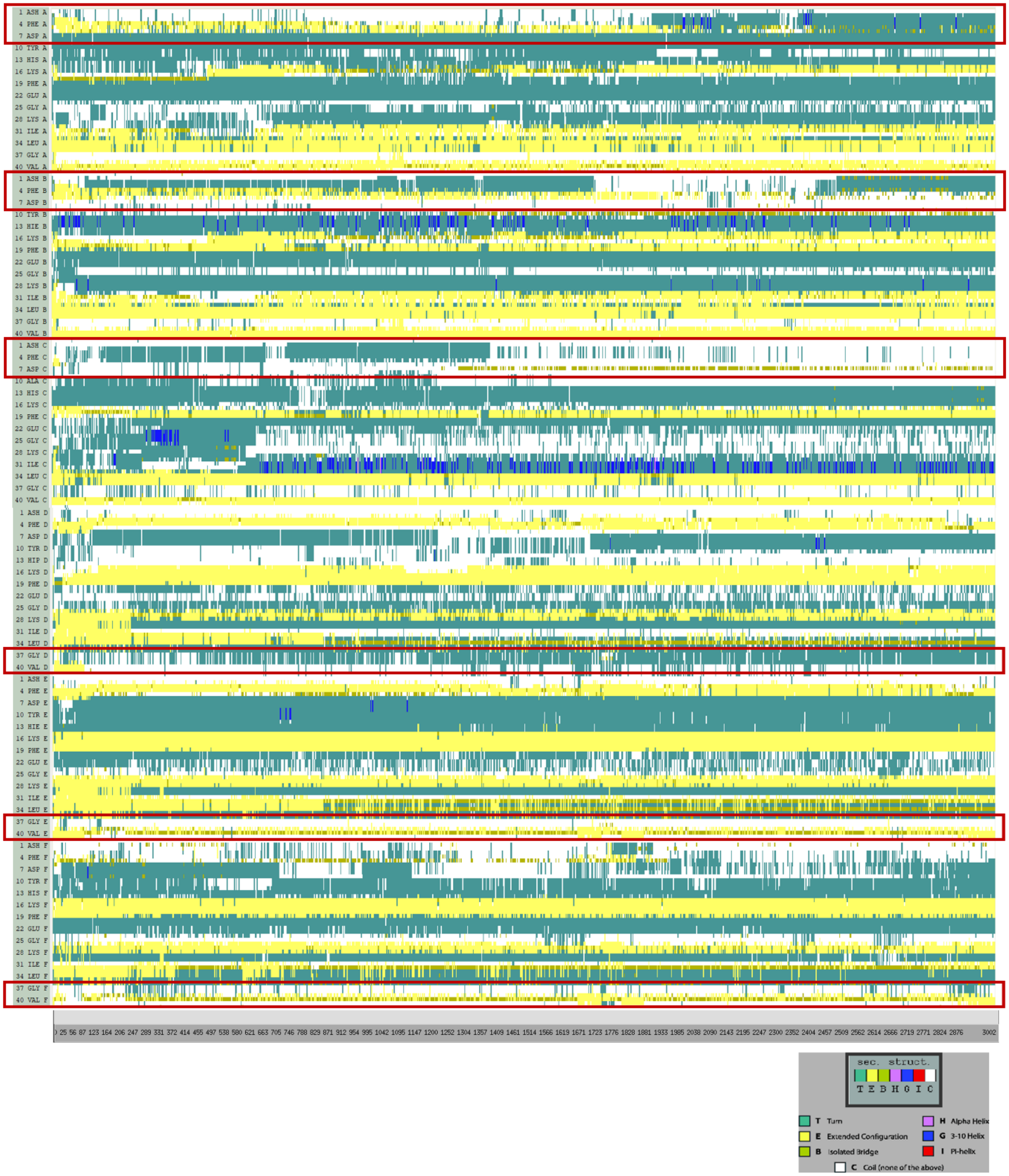

Supplementary Figure S6. Secondary structure timeline analysis during 300 ns MD simulation of (a) Aβ$_{1-42}$ Y10A mutant fibril (PDB ID: 2NAO) (a) unbound and (b) bound to cyclotide Cter-M.

Supplementary Table S7. Analysis of protein-protein interactions at t=0 ns (top) and t=300ns (bottom) frames between cyclotide Cter-M (PDB ID: 2LAM) and Y10A mutant form of A$\beta_{1\text{-}42}$ fibril (PDB ID: 2NAO).

**Interactions**	**[a]Maestro**	**[a]PPCheck**	**2NAO**				**2LAM**				**[b]Type of Bond**	**Distance Å**
			**Residue-1**				**Residue-2**					
			**Res No.**	**Res Name**	**Chain**	**Atom Name**	**Res No.**	**Res Name**	**Chain**	**Atom Name**		
**t=0 ns**												
Hydrogen Bond	✓	-	6	HIS	C	NE2	1	GLY	Z	O	SB	3.5
Aromatic Hydrogen Bond	✓	-	4	PHE	B	CE2	29	ASN	Z	O	SB	3.1
	✓	-	4	PHE	B	CZ	29	ASN	Z	O	SB	3.2
	✓	-	6	HIS	C	CE1	1	GLY	Z	O	SB	4.2
Hydrophobic Interactions	-	✓	12	VAL	A	CB	2	LEU	Z	CB	SS	6.2
	-	✓	40	VAL	E	CB	15	TYR	Z	CB	SS	6.5
	-	✓	40	VAL	E	CB	16	VAL	Z	CB	SS	6.9
	-	✓	40	VAL	F	CB	15	TYR	Z	CB	SS	4.9
Electrostatic Interactions	-	✓	14	HIS	B	CB	7	GLU	Z	CB	SS	9.3
	-	✓	13	HIS	C	CB	7	GLU	Z	CB	SS	9.3
	-	✓	14	HIS	C	CB	7	GLU	Z	CB	SS	7.4
**t=300 ns**												
Hydrogen Bond	✓	-	12	VAL	A	O	4	THR	Z	OG1	BS	2.6
	-	✓	14	HIS	C	NE2	7	GLU	Z	OE2	SS	3.3
Aromatic Hydrogen Bond	✓	-	4	PHE	B	CZ	1	GLY	Z	O	SB	3.7
	✓	-	38	GLY	F	O	15	TYR	Z	CE2	BS	3.3
Hydrophobic Interactions	-	✓	12	VAL	A	CB	2	LEU	Z	CB	SS	5.2
	-	✓	4	PHE	C	CB	25	ILE	Z	CB	SS	4.9
	-	✓	40	VAL	D	CB	15	TYR	Z	CB	SS	6.3
	-	✓	40	VAL	D	CB	16	VAL	Z	CB	SS	5.5
	-	✓	41	ILE	D	CB	16	VAL	Z	CB	SS	6.8
Electrostatic Interactions	-	✓	13	HIS	B	CB	7	GLU	Z	CB	SS	4.8
	-	✓	6	HIS	C	CB	7	GLU	Z	CB	SS	8.6
	-	✓	13	HIS	C	CB	7	GLU	Z	CB	SS	9.2
	-	✓	14	HIS	C	CB	7	GLU	Z	CB	SS	8.1

[a] Protein-protein interaction identified from Maestro and/or PPCheck is highlighted with ✓.
[b] "SS" represents sidechain-sidechain interaction, "SB" represents sidechain-backbone interaction, and "BB" represents backbone-backbone mode of interaction between the two interacting amino acids.
